# ATP7B R778L mutant hepatocytes resist copper toxicity by activating autophagy and inhibiting necroptosis

**DOI:** 10.1038/s41420-023-01641-5

**Published:** 2023-09-16

**Authors:** Shan Tang, Chen Liang, Wei Hou, Zhongjie Hu, Xinyue Chen, Jing Zhao, Wei Zhang, Zhongping Duan, Li Bai, Sujun Zheng

**Affiliations:** 1 The First Unit, Department of Hepatology, Beijing YouAn Hospital, Capital Medical University, Beijing, China; 2 The Fourth Unit, Department of Hepatology, Beijing YouAn Hospital, Capital Medical University, Beijing, China; 3 Beijing Key Laboratory of Liver Failure and Artificial Liver Treatment Research, Beijing, China

**Keywords:** Autophagy, Pathogenesis, Liver diseases

## Abstract

Wilson’s disease (WD) is an inherited disease characterized by copper metabolism disorder caused by mutations in the adenosine triphosphatase copper transporting β gene (ATP7B). Currently, WD cell and animal model targeting the most common R778L mutation in Asia is lacking. In addition, the mechanisms by which hepatocytes resist copper toxicity remain to be further elucidated. In this study, we aimed to construct a novel WD cell model with R778L mutation and dissected the molecular basics of copper resistance. A novel HepG2 cell line stably expressing the ATP7B R778L gene (R778L cell) was constructed. The expression of necroptosis- and autophagy-related molecules was detected by PCR and Western blot (WB) in wild-type (WT) HepG2 and R778L cells with or without CuSO_4_ treatment. In addition, we detected and compared the levels of autophagy and necroptosis in CuSO_4_-treated R778L cells with the activation and inhibition of autophagy. Moreover, the mRNA and protein levels of autophagy and necroptosis signaling molecules were compared in R778L cells with the overexpression and knockdown of Unc-51 Like Autophagy Activating Kinase 1 (ULK1) and Autophagy Related 16 Like 1 (ATG16L1). We successfully constructed an R778L mutation HepG2 cell line. CuSO_4_ triggered the enhanced expression of autophagy and necroptosis signaling molecules in WT HepG2 cells and R778L cells. Remarkably, higher levels of autophagy and necroptosis were observed in R778L cells compared with those in WT cells. Autophagy activation led to weakened necroptosis mediated by RIPK3 and MLKL, conversely, autophagy inhibition brought about enhanced necroptosis. At the molecular level, ULK1- and ATG16L1 overexpression resulted in reduced necroptosis levels and vice versa. ULK1- and ATG16L1-mediated autophagy activation protects hepatocytes against RIPK3- and MLKL-mediated necroptosis in our new WD cell model treated with CuSO_4_. Targeted therapy by autophagy activation or necroptosis inhibition may be a novel and effective strategy to treat WD.

## Introduction

Wilson disease (WD) is an autosomal recessive disorder of copper metabolism due to mutations in the ATP7B gene that encodes copper-transporting P-type ATPase [1]. WD is not rare as we imagined, with the estimated prevalence of symptomatic disease at 1 in 30,000 [2]. The prevalence is believed to be higher in China than in Western countries [3]. Until now, more than 800 ATP7B mutations have been identified. The mutations in ATP7B disrupt Cu homeostasis and lead to the accumulation of copper in the liver and other tissues [4]. Excessive copper leads to a variety of clinical presentations, including neurologic symptoms, hepatitis, cirrhosis, acute or chronic liver failure, or psychiatric manifestations [5]. Once the diagnosis is established, WD patients should be treated lifelong, or it will be fatal, with a mortality of about 5.0–6.1%. Current treatments include pharmacological therapies and liver transplantation. Chelators and zinc salts are the most common treatment drugs for WD. Chelators function by increasing the urinary excretion of copper; however, they are associated with numerous severe side effects, which occur in approximately 30% of WD patients. Zinc salts can decrease copper uptake from the gastrointestinal tract [6], nevertheless, the symptoms of WD may not be well-controlled by them [7]. Besides, liver transplantation faces challenges such as the shortage of donor liver and a high rate of complications. In this context, it is essential to dissect the molecular mechanisms of WD from a novel perspective in order to explore new effective treatment strategies.

The point mutation R778L in exon 8 is the most common ATP7B mutation in WD patients from Asia, with an allele frequency of 12–39% [8]. It has been reported that R778L mutation is associated with severe clinical symptoms [9]. Considering the current lack of WD cell and animal models aimed at R778L mutation, it is urgently needed for scientists engaged in WD research to address this issue.

As we all know, Cu toxicity is the central link of WD. In this setting, dissecting the mechanisms by which hepatocytes counteract Cu toxicity is of great importance because it opens up the opportunity for new targeted therapies. Both autophagy and necroptosis are stress responses governing the ultimate fate of a cell. Autophagy is an evolutionarily conserved cellular degradation process [10], which plays a critical role in cellular homeostasis through degrading proteins, glycogen, lipids, and organelles and further provides nutrients and biomolecular building blocks for cell survival [11, 12].

Necroptosis is a newly discovered pathway of regulated necrosis [13], which is mediated by receptor-interacting protein kinase 1 (RIPK1), RIPK3, and mixed-lineage kinase domain-like pseudokinase (MLKL). When caspase-8 is inhibited, RIPK1 interacts with RIPK3 to form a complex called ‘necrosome’ and initiates necroptosis [14].

The cumulative evidence has proved that both autophagy and necroptosis are closely associated with multiple liver diseases, such as drug-induced liver injury [15] and non-alcoholic fatty liver disease [16]. Nevertheless, current reports on the role of autophagy in WD are still controversial. Polishchuk et al. [17] demonstrated that autophagy is activated in response to copper overload to prevent copper-induced cell death in ATP7B-deficient hepatocytes. On the contrary, Pantoom et al. [18] believed that HepG2 cells deficient in ATP7B exhibit autophagy deficiency under high copper conditions. In terms of necroptosis, no studies have reported the role of necroptosis in WD currently. Remarkably, copper-containing nanomaterials have been documented recently to induce MLKL-mediated necroptosis in human gastric cancer cells [19].

The interplay between autophagy and necroptosis, especially in WD, needs to be elucidated. A recent study proposed that ULK1 acts as a regulator of RIPK1-mediated necroptosis [20], and deprivation of ULK1 enhances necroptosis. In addition, autophagy protein ATG16L1 has been documented to prevent necroptosis in the intestinal epithelium [21]. Therefore, necroptosis may act as a downstream signal of the autophagy pathway.

In this work, we first established the ATP7B R778L mutant human hepatocellular carcinoma HepG2 cell line and analyzed the effect of this mutation on the ATP7B protein. Then, we investigated the interplay between autophagy and necroptosis in WD and hypothesized that elevated autophagy suppresses necroptosis and thus resists copper toxicity in the ATP7B R778L mutant HepG2 cell line. To testify to this hypothesis, first, we determined the levels of autophagy and necroptosis in ATP7B R778L HepG2 cells with or without copper stimulation; second, we compared the degree of autophagy and necroptosis in R778L and wild-type HepG2 cells with copper stimulation; third, we investigated the regulation of autophagy on necroptosis by activating and inhibiting autophagy; lastly, we dissected the molecular basic by which autophagy regulates necroptosis. Altogether, our study highlighted that both autophagy and necroptosis are involved in the pathological mechanism of WD and suggested that activating autophagy or inhibiting necroptosis might be an ideal therapeutic strategy to treat WD.

## Results

### Overview of the ATP7B model and the effect of R778L mutation on the structure

The wild-type ATP7B protein was an unstable hydrophobic protein with an instability index (II) of 49.77 and a Grand average of hydropathicity (GRAVY) value of 0.111. The calculated aliphatic index (100.43) indicated the high thermal stability of the protein. The R778L mutation caused slight changes in the physico-chemical properties with elevated GRAVY, suggesting the increase in the hydrophobicity of the mutant protein (Supplementary Table S3). Replacing the positively charged Arg with the hydrophobic Leu was associated with the retention of ATP7B in the endoplasmic reticulum, indicative of at least partial misfolding (Supplementary Fig. S3). Analysis of the transmembrane structure showed that wild-type ATP7B had eight transmembrane helical structures. The R778L mutation was located in the S4 region; this mutation did not change the position of the transmembrane helix directly in the S4 region but changed the transmembrane helix of S2 and S6 (Supplementary Fig. S4). We then constructed an ATP7B protein model in order to analyze the effect of R778L mutation on protein structure more intuitively. The model structure encompassed the C-terminal segment of human ATP7B, which was composed of 486–1406 residues in the complete sequence of 1465 amino acid residues (Supplementary Fig. S5). It consisted of a TM domain formed by eight TM helices and a cytosolic portion, which represents a structural collection of nucleotide-bind (N)-, phosphorylation (P)-, actuator (A)-domains, and multiple metal-binding domains (MBD) 5, 6. The superposition of the R778L mutant ATP7B protein with the wild-type ATP7B three-dimensional structure made it clear that there were obvious changes in the transmembrane helical domain and nucleotide-bind domain (Supplementary Fig. S6). In the ATP7B model, Arg778 was located at TM4 in the vicinity of the conserved residues Leu730, Glu781, Gly988, and Ala990 (Supplementary Fig. S7). A charge balance in this region seemed to play an important role in maintaining the ATP7B folding and/or restricting protein motions. What’s more, the ATP7B model displayed a highly conserved protein core, with Arg778 located in the conserved region, suggesting that the R778L mutation would have a strong effect on protein function (Supplementary Fig. S8).

### Establishment of stable ATP7B R778L mutant HepG2 cell line (R778L cells)

**Fig. 1:**
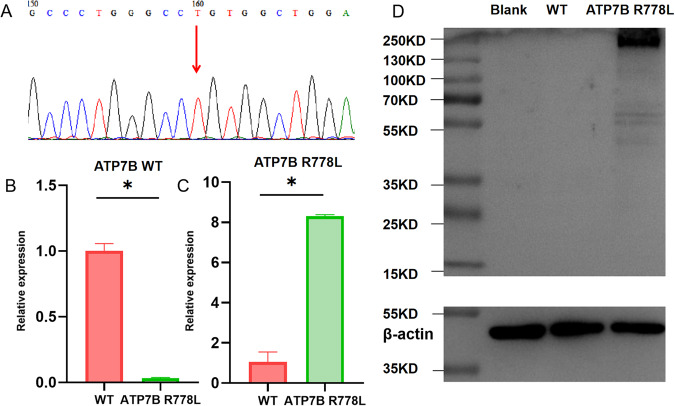
Establishment of a stable ATP7B R778L mutant HepG2 cell line. **A** The sequencing result in ATP7B R778L mutant HepG2 cell line. **B** The mRNA levels of ATP7B in wild type and ATP7B R778L mutant HepG2 cell line. **C** The mRNA levels of ATP7B R778L in wild type and ATP7B R778L mutant HepG2 cell line. **D** The protein expression of Flag detected by western blot. *P < 0.05. WT, wild type.

HepG2 cells with ATP7B R778L mutation were constructed and then screened with 2 μg/ml puromycin. The sequencing results showed that the 2333 nucleotide of the mutant ATP7B was T, indicating that the mutation in 2333 nucleotide was successful (Fig. 1A). The mRNA levels of WT and R778L ATP7B were measured by qPCR. The results showed that the expression of ATP7B WT mRNA was decreased (Fig. 1B), but the expression of ATP7B R778L mRNA was increased (Fig. 1C) in ATP7B R778L mutant stable cell lines, suggesting that HepG2 cell lines with ATP7B R778L mutation were constructed successfully. Western blot detection displayed Flag was positive, supporting the successful construct of R778L cells (Fig. 1D).

### Copper overload triggers autophagy and necroptosis in R778L cells

**Fig. 2:**
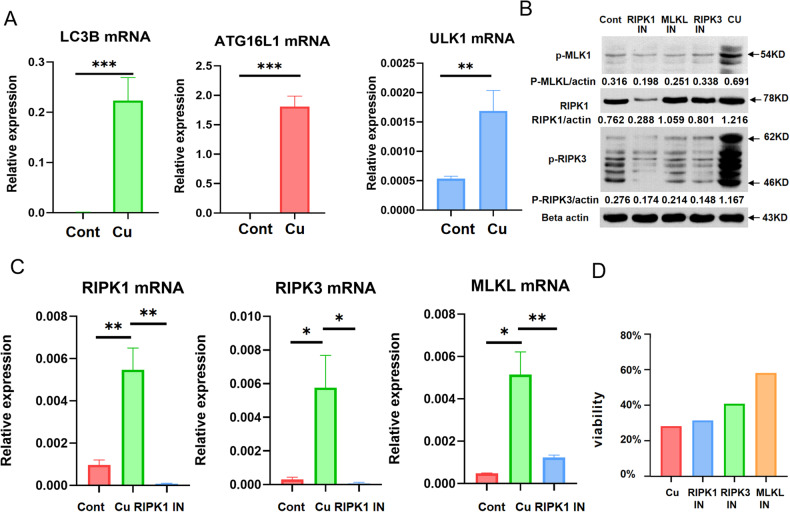
Copper overload triggers autophagy and necroptosis in ATP7B R778L mutant HepG2 cells. **A** The mRNA levels of autophagy markers, including LC3B, ATG16L1, and ULK1, in ATP7B R778L mutant HepG2 cell line treated with CuSO_4_. **B** The protein levels of necroptosis markers (RIPK1, RIPK3, and MLKL) in ATP7B R778L mutant HepG2 cells treated by CuSO_4_ with or without necroptosis inhibitor. **C** The mRNA levels of necroptosis markers (RIPK1, RIPK3, and MLKL) in ATP7B R778L mutant HepG2 cells treated by CuSO_4_ with or without necroptosis inhibitor. **D** The cell viability of ATP7B R778L mutant HepG2 cells treated by CuSO_4_ with or without necroptosis inhibitor. *P < 0.05, **P < 0.01, ***P < 0.001. cont, ATP7B R778L mutant HepG2 cells without copper treatment; cu, ATP7B R778L mutant HepG2 cells with copper treatment; RIPK1 IN, ATP7B R778L mutant HepG2 cells treated by CuSO_4_ and RIPK1 inhibitor; RIPK3 IN, ATP7B R778L mutant HepG2 cells treated by CuSO_4_ and RIPK3 inhibitor; MLKL IN, ATP7B R778L mutant HepG2 cells treated by CuSO_4_ and MLKL inhibitor.

To investigate the effect of copper overload on autophagy in R778L cells, we compared the expression of autophagy-related makers in R778L cells with or without CuSO_4_ treatment. Quantitative PCR analysis showed that the mRNA levels of autophagy-related markers, including LC3B, ATG16L1, and ULK1, were obviously upregulated in CuSO_4_-treated R778L cells (Fig. 2A). These observations indicated that copper overload triggers autophagy in R778L cells. To determine the role of necroptosis in WD, we first compared the levels of necroptosis-related signaling molecules in the liver tissues of ATP7B-/- mice and wild-type controls. By analyzing the gene data of GSE125637, 527 DEGs were screened. Especially, the expression levels of MLKL in the liver tissues of ATP7B-/- mice were significantly higher than those in the livers of wild-type controls (Supplementary Fig. S9). Then, the mRNA and protein levels of signaling molecules related to necroptosis were compared in CuSO_4_-treated R778L cells with or without necroptosis inhibitor treatment. Real-time PCR and WB analyses revealed that the protein and mRNA levels of RIPK1, RIPK3, and MLKL were remarkably upregulated in CuSO_4_-treated R778L cells but obviously downregulated after RIPK1, RIPK3 or MLKL inhibitor treatment (Fig. 2B, C). To further confirm the pivotal role of necroptosis in CuSO_4_-induced cell toxicity, we analyzed the effect of necroptosis inhibitors on the viability of cells treated by CuSO_4_. The CCK8 assay showed that the viability of R778L cells was evidently decreased after treatment with CuSO_4_. Importantly, the cell viability was remarkably elevated after necroptosis inhibitor treatment (Fig. 2D). That is to say, the cytotoxicity induced by CuSO_4_ may be weakened by necroptosis inhibitors (especially MLKL inhibitor) in R778L cells. Therefore, copper overload triggers autophagy and necroptosis in R778L cells.

### Copper overload triggers higher levels of autophagy and necroptosis in R778L cells compared with WT cells

**Fig. 3:**
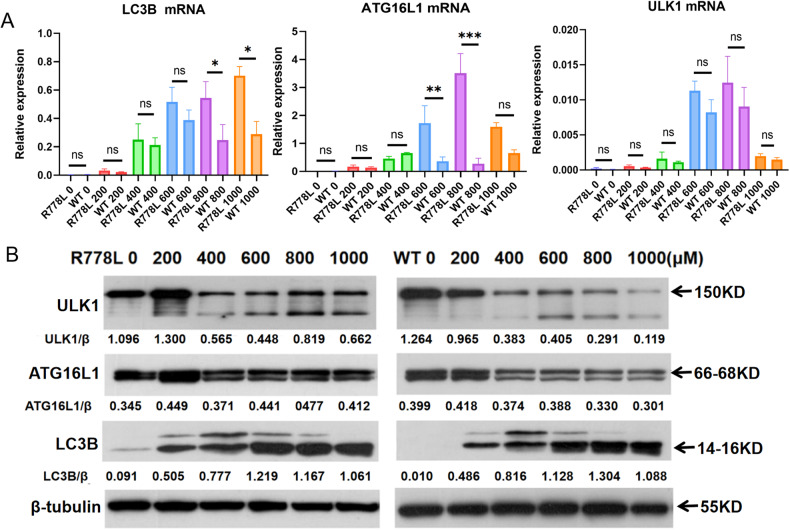
ATP7B R778L mutant HepG2 cells exhibit higher levels of autophagy than WT HepG2 cells. **A** The mRNA levels of autophagy markers, including LC3B, ATG16L1, and ULK1, in ATP7B R778L mutant and wild-type HepG2 cell line treated with copper. **B** The protein levels of autophagy markers, including LC3B, ATG16L1, and p-ULK1, in ATP7B R778L mutant and wild-type HepG2 cell line treated with copper. R778L, ATP7B R778L mutant HepG2 cell line; WT, wild-type HepG2 cell line.

**Fig. 4:**
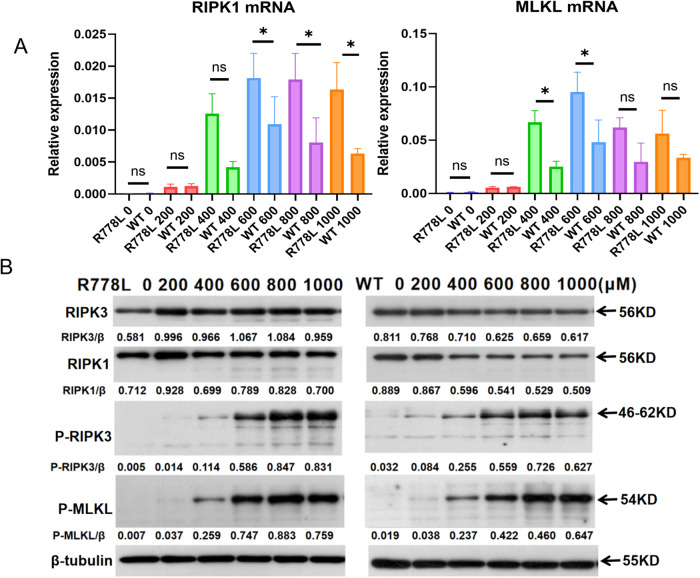
ATP7B R778L mutant HepG2 cells exhibit higher levels of necroptosis than in WT HepG2 cells. **A** The mRNA levels of necroptosis markers, including RIPK1 and MLKL, in ATP7B R778L mutant and wild-type HepG2 cell line treated with copper. **B** The protein levels of necroptosis markers, including RIPK1, RIPK3, p-RIPK3, and p-MLKL, in ATP7B R778L mutant and wild-type HepG2 cell line treated with copper. R778L, ATP7B R778L mutant HepG2 cell line; WT, wild-type HepG2 cell line.

To examine whether ATP7B deficiency contributes to autophagy and necroptosis, we analyzed and compared the autophagy and necroptosis levels in R778L and WT cells treated with CuSO_4_ at concentrations ranging from 200 to 1000 μM. Quantitative PCR analysis showed that the mRNA levels of autophagy markers, including LC3B, ATG16L1, ULK1, and necroptosis markers, including RIPK1 and MLKL, were obviously higher or exhibited an elevated tendency in CuSO_4_-treated R778L cells compared to those in WT cells (Figs. 3A and 4A). Similarly, the protein expression of autophagy markers (especially ATG16L1 and ULK1) and necroptosis markers, including RIPK1, RIPK3, MLKL, and p-MLKL, was also markedly enhanced in R778L cells, as detected by western blot (Figs. 3B and 4B). Collectively, R778L cells exhibit stronger activation of autophagy and necroptosis faced with the pressure of copper overload, compared with WT cells.

### Autophagy activation inhibits necroptosis

**Fig. 5:**
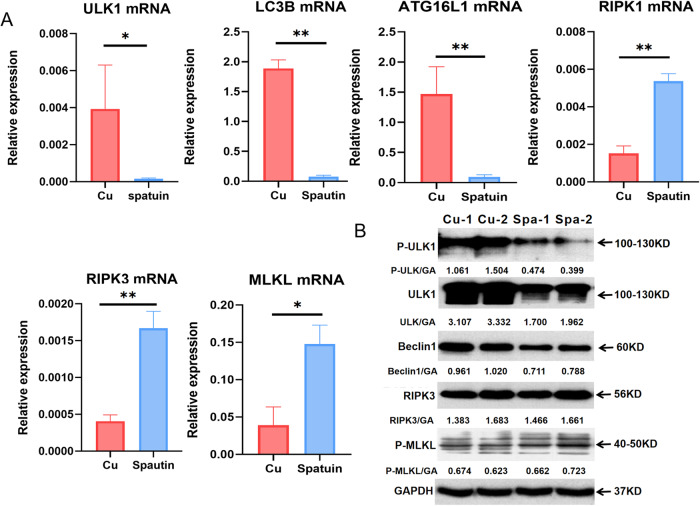
Autophagy inhibition enhances necroptosis. **A** The mRNA levels of autophagy markers (LC3B, ATG16L1, and ULK1) and necroptosis markers (RIPK1, RIPK3, and MLKL) in ATP7B R778L mutant HepG2 cell line with or without spautin-1 treatment. **B** The protein levels of autophagy markers (ULK1, P-ULK1, and Beclin1) and necroptosis markers (RIPK3 and p-MLKL) in ATP7B R778L mutant HepG2 cell line with or without spautin-1 treatment. *P < 0.05, **P < 0.01. Spa, spautin-1.

**Fig. 6:**
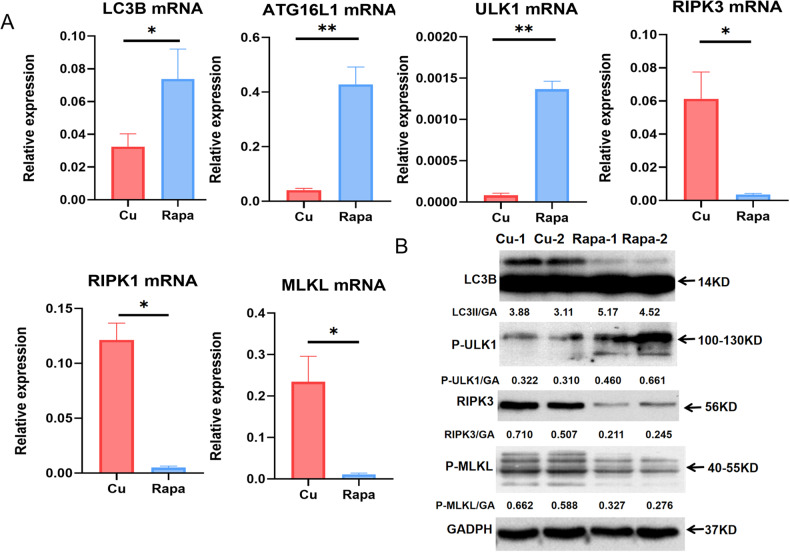
Autophagy activation inhibits necroptosis. **A** The mRNA levels of autophagy markers (LC3B, ATG16L1, and ULK1) and necroptosis markers (RIPK1, RIPK3, and MLKL) in ATP7B R778L mutant HepG2 cell line with or without spautin-1 treatment. **B** The protein levels of autophagy markers (LC3B and P-ULK1) and necroptosis markers (RIPK3 and p-MLKL) in ATP7B R778L mutant HepG2 cell line with or without rapamycin treatment. *P < 0.05, **P < 0.01. Rapa, rapamycin.

As demonstrated above, the copper overload induces autophagy and necroptosis, and higher levels of autophagy and necroptosis were observed in R778L cells than in WT cells. However, the crosstalk between autophagy and necroptosis remains to be elucidated. To provide substantive evidence for the close relationship between autophagy and necroptosis, we conducted autophagy activation and inhibition experiments in R778L cells. First, we examined the effects of spautin-1, a specific inhibitor of autophagy, on the activation of autophagy and necroptosis induced by CuSO_4_. As a result, spautin-1 significantly reduced the gene levels of LC3B, ULK1, and ATG16L1 activated by CuSO_4_ but promoted the expression of necroptosis-related markers, including RIPK1, RIPK3, and MLKL (Fig. 5A). WB results revealed markedly decreased protein levels of ULK1, p-ULK1, and Beclin1 but increased expression of p-MLKL (Fig. 5B). In addition, we compared the ratio between autophagy and necroptosis in the CuSO_4_-treated cells with or without spautin-1 treatment. The results showed that the ratios of autophagy to necroptosis were significantly lower in spautin-1-treated cells at the mRNA and protein levels, supporting that the balance between autophagy and necroptosis shifted toward necroptosis after autophagy inhibition (Supplementary Fig. S10). On the other hand, we assessed the effect of rapamycin, a classic autophagy activator, on the levels of autophagy and necroptosis in CuSO_4_-treated R778L cells. As a result, the administration of rapamycin led to significantly elevated levels of autophagy in R778L cells treated with CuSO_4_, as manifested by increased expression of LC3B, ULK1, and ATG16L1. On the contrary, CuSO_4_-induced necroptosis was obviously attenuated by rapamycin, as evidenced by decreased expression of RIPK1, RIPK3, and MLKL (Fig. 6A). At the protein levels, the expression of autophagy markers LC3B and p-ULK1 was markedly enhanced in rapamycin-treated cells, but the levels of necroptosis markers RIPK3 and p-MLKL were significantly abated (Fig. 6B). We also assessed and compared the ratios of autophagy to necroptosis in the CuSO_4_-treated cells with or without rapamycin treatment. The data showed that the ratios of autophagy to necroptosis were significantly upregulated in rapamycin-treated cells both at the mRNA and protein levels (Supplementary Fig. S11). Altogether, autophagy activation protects against copper-induced cytotoxicity by suppressing necroptosis.

### The inhibition of necroptosis by autophagy was mediated via ULK1 and ATG16L1

**Fig. 7:**
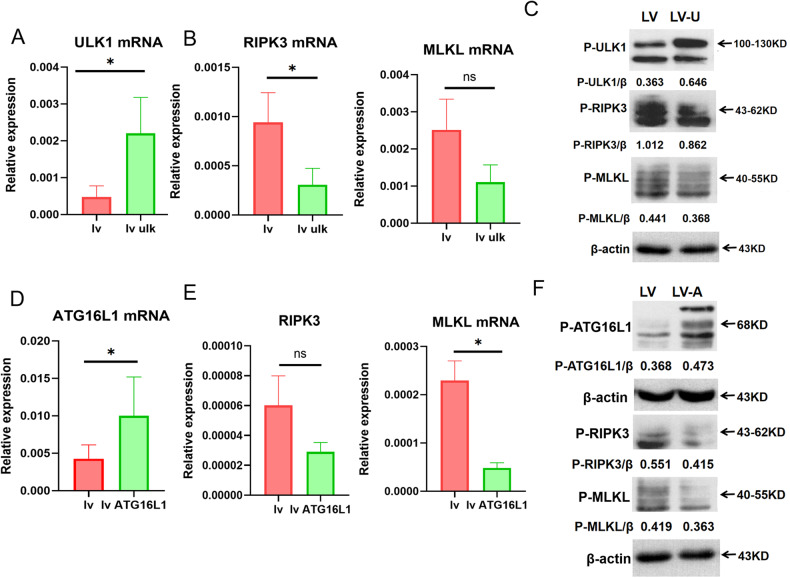
Overexpression of ULK1 and ATG16L1 inhibits necroptosis. **A** The mRNA levels of ULK1 in ATP7B R778L-mutant HepG2 cell line transfected with ULK1-overexpressed lentiviruses. **B** The mRNA levels of necroptosis markers (RIPK3 and MLKL). **C** The protein levels of p-ULK1 and necroptosis markers (p-RIPK3 and p-MLKL) in ATP7B R778L-mutant HepG2 cell line transfected with ULK1-overexpressed lentiviruses. **D** The mRNA levels of ATG16L1 and necroptosis markers (RIPK3 and MLKL) in ATP7B R778L-mutant HepG2 cell line transfected with ATG16L1-overexpressed lentiviruses. **E** The mRNA levels of necroptosis markers (RIPK3 and MLKL) in ATP7B R778L-mutant HepG2 cell line transfected with ATG16L1-overexpressed lentiviruses. **F** The protein levels of p-ATG16L1 and necroptosis markers (p-RIPK3 and p-MLKL) in ATP7B R778L-mutant HepG2 cell line transfected with ATG16L1-overexpressed lentiviruses.

**Fig. 8:**
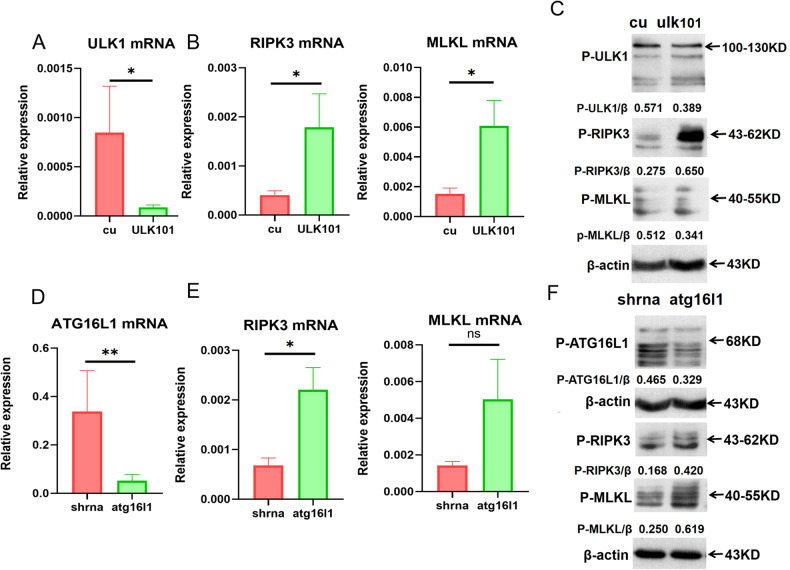
Knockdown of ULK1 and ATG16L1 enhances necroptosis. **A** The mRNA levels of ULK1 in ATP7B R778L-mutant HepG2 cell line treated with ULK inhibitor-ULK101. **B** The mRNA levels of necroptosis markers (RIPK3 and MLKL) in ATP7B R778L-mutant HepG2 cell line treated with ULK inhibitor-ULK101. **C** The protein levels of p-ULK1 and necroptosis markers (p-RIPK3 and p-MLKL) in ATP7B R778L-mutant HepG2 cell line treated with ULK101. **D** The mRNA levels of ATG16L1 in ATP7B R778L-mutant HepG2 cell line transfected with lentivirus-enveloped shATG16L1. **E** The mRNA levels of necroptosis markers (RIPK3 and MLKL) in ATP7B R778L-mutant HepG2 cell line transfected with lentivirus-enveloped shATG16L1. **F** The protein levels of p-ATG16L1 and necroptosis markers (p-RIPK3 and p-MLKL) in ATP7B R778L-mutant HepG2 cell line transfected with lentivirus-enveloped shATG16L1.

Finally, we investigated the molecular mechanisms by which autophagy inhibits necroptosis in R778L cells and focused on ULK1 and ATG16L1. To this end, ULK1- and ATG16L1-overexpressed lentiviruses were transferred into R778L cells. As a result, the transfer brought about the elevated expression of ULK1 and ATG16L1, respectively, at the gene and protein levels (Fig. 7a, c, d, f). Remarkably, reduced gene and protein levels of necroptosis-related markers, including RIPK3 and MLKL, were noticed in the ULK1 and ATG16L1-overexpressed cells (Fig. 7b, c, e, f). Moreover, we analyzed the ratios of ULK1 and ATG16L1 to necroptosis-related markers. Higher ratios were noticed in R778L cells transferred with ULK1 or ATG16L1-overexpressed lentiviruses, suggesting that the balance between autophagy and necroptosis shifted toward autophagy in the setting of ULK1 or ATG16L1 overexpression (Supplementary Fig. S12). On the other hand, we treated R778L cells with ULK101, a specific ULK1 inhibitor, followed by copper stimulation. As expected, decreased expression of ULK1 but enhanced levels of RIPK3 and MLKL were observed in ULK101-treated cells (Fig. 8a–c). Similarly, the expression of ATG16L1 was downregulated after transferring lentivirus-enveloped shATG16L1 into R778L cells (Fig. 8d, f). On the contrary, the levels of RIPK3 and MLKL detected by real-time PCR, as well as the protein expression of p-RIPK3 and p-MLKL detected by western blot, were enhanced in R778L cells treated with shATG16L1 (Fig. 8e, f). We also analyzed the ratios between autophagy- and necroptosis-related markers at gene and protein levels. The ratios were decreased when treated with ULK101 or lentivirus-enveloped shATG16L1 (Supplementary Fig. S13), suggesting the balance between autophagy and necroptosis shifted toward necroptosis in the setting of ULK1 or ATG16L1 inhibition. Therefore, autophagy signaling molecules, including ULK1 and ATG16L1, mediate the inhibition of necroptosis.

## Discussion

In the present work, we constructed a novel WD cell model with ATP7B R778L mutation. In this setting, we demonstrated: (1) both autophagy and necroptosis are activated in WD; (2) higher levels of autophagy and necroptosis are triggered in R778L cells compared with WT HepG2 cells; (3) autophagy activation inhibits necroptosis; and (4) autophagy inhibits necroptosis via ULK1 and ATG16L1. To the best of our knowledge, this is the first time to elucidate the protective mechanism of autophagy against copper toxicity using a novel WD cell model from a new perspective, namely, autophagy-mediated inhibition of necroptosis.

The ATP7B R778L mutation is the most common mutation site in Asian WD patients. To predict the effect of ATP7B R778L gene mutation on the structure and function of ATP7B protein, we constructed the ATP7B protein model. The analysis result demonstrated that the R778L mutation has a great effect on the structure and function of the ATP7B protein. This data may provide, at least in part, a possible explanation for the phenomenon that patients with R778L mutation often manifest severe clinical symptoms. Considering the high prevalence and importance of R778L mutation for Asian WD patients, we next constructed successfully a novel WD cell model with ATP7B R778L gene mutation. As far as our information goes, this is the first HepG2 cell model that can stably express the R778L mutant ATP7B gene. Subsequent experiments were performed in the setting of this new cell model.

The liver is a highly dynamic metabolic organ. Autophagy is a cellular catabolic process mediated by the lysosome, which plays a crucial role in maintaining cellular and metabolic homeostasis in the liver. Dysfunction of autophagy has been linked to multiple liver diseases. In terms of WD, the function of autophagy in WD is still complex and controversial. Recent data highlight the protective role of autophagy against copper toxicity during WD. Activation of autophagy was observed in the livers of WD patients and animal models and has been confirmed to protect hepatocytes from cell apoptosis [17]. In contrast, the latest work demonstrated that Gandouling alleviates cognitive dysfunction in WD by reducing autophagy and oxidative stress [22]. In the present work, the gene and protein expression of signaling molecules related to autophagy was obviously elevated in ATP7B R778L mutant and wild-type HepG2 cells after treatment with CuSO_4_. Especially, the levels of autophagy were remarkably higher in R778L cells as compared with those in WT cells, as manifested by more elevated gene and protein expression of autophagy-related molecules in the former. Thus, CuSO_4_ treatment induces autophagy activation in R778L and WT cells, especially in R778L cells. This is the first work to analyze the autophagy levels in hepatocytes with ATP7B R778L mutation upon copper treatment. Importantly, we demonstrated that the levels of autophagy activation are significantly higher in R778L cells than those in WT cells.

Necroptosis, as a novel cell death mode, has been confirmed to be critically involved in diverse liver diseases. However, whether necroptosis plays a beneficial or harmful role in liver disease is environmental- and cell-dependent. In the case of hepatic fibrosis, hepatocyte necroptosis was reported to promote the development of hepatic fibrosis [23], whereas necroptosis in hepatic stellate cells was confirmed to promote fibrosis resolution [24]. Unfortunately, no work has investigated the possible role of necroptosis in WD so far. Our results showed that necroptosis is activated in CuSO_4_-treated HepG2 cells, which can be obviously inhibited by the administration of specific necroptosis inhibitors. The inhibition of necroptosis is accompanied by the elevation in cell viability, supporting the detrimental effect of necroptosis on HepG2 cells. Of note, the levels of necroptosis are higher in R778L cells compared with WT cells, as shown by boosted expression of necroptosis-related signaling molecules in the former. Our data provide the first-line evidence for exploring the potential function of necroptosis in WD.

The balance between cell death and survival is essential for maintaining homeostasis and preventing disease. The coexistence of autophagy and cell death has been widely revealed, and the interplay between autophagy and necroptosis is also being investigated in depth. The ultimate fate of a cell upon stress is determined by the integration of different cellular stress responses [25]. According to our data, the levels of both autophagy and necroptosis were markedly enhanced in R778L cells compared with those in WT cells. To further investigate the interaction between these two stress responses in our WD cell model, we conducted autophagy activation and inhibition experiments and analyzed the effects of autophagy activation and inhibition on the levels of necroptosis. After treatment with rapamycin, R778L cells stimulated by CuSO_4_ exhibited stronger autophagy and weaker necroptosis, as manifested by elevated levels of autophagy-related markers but reduced expression of necroptosis-related signaling molecules at the mRNA and protein levels. Moreover, the ratio of autophagy to necroptosis was significantly upregulated, which provides further support for the enhanced autophagy and weakened necroptosis in the setting of rapamycin stimulation. On the contrary, the administration of spautin-1 led to decreased autophagy but increased necroptosis levels. The balance between autophagy and necroptosis was moved toward necroptosis. Therefore, autophagy activation inhibits necroptosis in R778L cells; that is to say, autophagy exerts a beneficial protective effect by inhibiting necroptosis signaling in R778L cells. To the best of our knowledge, this is the first work to elucidate the protective role of autophagy in WD from a novel perspective, namely, autophagy-mediated inhibition of necroptosis signaling. Our finding is partially consistent with recent reports by Altman et al., who confirmed that human cytomegalovirus-induced autophagy prevents necroptosis in infected monocytes [26], and Zhang et al., who demonstrated that impaired autophagy-mediated necroptosis signaling activation mechanistically contributed to a loss of cardiomyocytes, adverse ventricular remodeling and progressive heart failure after myocardial Infarction [27]. In contrast, the latest work documented that MLKL contributes to Western diet-induced liver injury through inhibition of autophagy and induction of necroptosis [28].

So, how does autophagy regulate necroptosis signaling at the molecular level in our ATP7B R778L WD cell model? The outstanding work by Wu et al. revealed that the autophagy-inducing kinase ULK1 phosphorylates the necroptosis-regulating kinase RIPK1, reduces the assembly of the death-inducing complex IIb/necrosome, and thereby inhibits RIPK1-mediated cell death [20]. Herein, RIPK1 functions as a substrate of ULK1. Moreover, autophagy protein ATG16L1 was confirmed to protect the intestinal epithelium from necroptosis, and survival can be improved by blocking RIPK1, RIPK3, or MLKL in ATG16L1^ΔIEC^ organoids in the presence of TNF-α [21]. In light of this, we next dissected the molecular mechanism by which autophagy inhibits necroptosis and focused on ULK1 and ATG16L1. According to our data, overexpression of ULK1 and ATG16L1 inhibits obviously necroptosis, as evidenced by reduced levels of RIPK3 or MLKL mRNA as well as p-RIPK3 and p-MLKL proteins in ULK1- and ATG16L1-overexpressed R778L cells. The ratios of ULK1 or ATG16L1 to necroptosis were significantly elevated in the setting of ULK1 and ATG16L1 overexpression. In contrast, knockdown of ULK1 and ATG16L1 promotes necroptosis, as shown by elevated levels of RIPK3 and/or MLKL mRNA as well as p-RIPK3 and/or p-MLKL proteins in R778L cells with shULK1- and shATG16L1 treatment. The balance between ULK1/ ATG16L1 and necroptosis was moved toward necroptosis in the setting of ULK1 and ATG16L1 knockdown. Together, ULK- and ATG16L1-mediated autophagy inhibits RIPK3- and MLKL-mediated necroptosis. As far as we know, this is the first study to elucidate the molecular mechanism by which autophagy inhibits necroptosis in WD.

In summary, this work developed a novel WD cell model with a special mutation in R778L. More importantly, we demonstrated that both autophagy and necroptosis are critically involved in the development of WD, and ULK1- and ATG16L1-mediated autophagy inhibits RIPK3- and MLKL-mediated necroptosis using this cell model.

## Conclusion

In the present work, we provided compelling evidence in favor of a prosurvival function of autophagy in WD. Through the new WD cell model, we proved autophagy activation could protect hepatocytes against RIPK3- and MLKL-mediated necroptosis. Our work provides new insights into the pathogenesis and therapeutic strategy (autophagy activation or necroptosis inhibition) of WD in the Asian population.

## Methods

### ATP7B protein model construction and analysis

The fold recognition server Phyre2 (http://www.sbg.bio.ic.ac.uk/phyre2/html/page.cgi?id=index) was used to identify the best structural template and domain of ATP7B protein, and molecular graphics were analyzed by Pymol software. ProtParam (http://www.sbg.bio.ic.ac.uk/phyre2/html/page.cgi?id=index) and Protscale (http://web.expasy.org/protscale) were used to predict the Physico-chemical properties and hydrophobic of amino acid residues in ATP7B wild-type and R778L mutation type protein, respectively. The ATP7B evolutionary conservation scores were computed via the ConSurf webserver (http://consurf.tau.ac.il/38).

### Cell culture

HepG2 cells (ATCC: HB-8065) were cultured in Dulbecco’s Modified Eagle Medium (DMEM) (Biochrom AG, Berlin, Germany) supplemented with 10% fetal bovine serum (Biochrom AG) and 2 mM L-Glutamine (PAA Laboratories GmbH, Pasching, Austria) at 37 °C under 5% CO_2_ in a humidified incubator. ATP7B R778L HepG2 cells were grown in DMEM supplemented with 10% fetal bovine serum, 2 mM L-Glutamine, and 2ug/ml puromycin (Invitrogen, Grand Island, NY, USA).

### Construction of ATP7B R778L overexpressed vectors

cDNA fragments coding for the N-terminal (ATP7B R778LN) and C-terminal (ATP7B R778LC) halves of mutant human ATP7B R778L (GenBank, NM_000053.3) were obtained by PCR amplification using the ‘primer 1’ for ATP7B R778LN, and ‘primer 2’ for ATP7B R778LC, respectively. The FLAG tag was amplified using the ‘primer 3’. The PCR was run under the following conditions: 98 °C for 3 min, 30 cycles of 98 °C for 30 s, 55 °C for 15 s, and 72 °C for 1 min with a final extension at 72 °C for 10 min. The lentiviral vector pLVX-CMV7-MCS-EF1a-Puro (Supplementary Fig. S1a) was linearized through restriction enzyme digestion by EcoRI-HF and XhoI. The final PCR products composed of ATP7B R778L and FLAG tag were cloned into the pLVX-CMV7-MCS-EF1a-Puro using a seamless cloning reaction system (Sangon Biotech, Shanghai, China) (Supplementary Fig. S1b) and then validated by DNA sequencing.

### Establishment of ATP7B R778L mutant stable HepG2 cell line (R778L cells)

The establishment of lentiviral constructs with ATP7B R778L mutation mainly included packaging, concentration, and purification. HEK-293T cells were transfected with plasmid vectors carrying ATP7B R778L and packaging plasmids pCMV-dR8.9 (Addgene, Cambridge, America) and pCMV-VSV-G (Addgene, Cambridge, America). Then, the packaged recombinant lentiviruses were harvested from the supernatant of post-infection cell cultures. The viral load of recombinant lentivirus was quantified in copies/ml by real-time PCR. Then, successfully established ATP7B R778L mutant lentivirus constructs were utilized to infect HepG2 cells seeded in a 6-well plate. The stable HepG2 cells overexpressing R778L ATP7B were obtained by continuous screening with 2 µg/mL puromycin for 2 weeks and checked by PCR and western blot analyses.

### Construction of pLVX-hULK1/-hATG16L1-3flag-ZsGreen-Bsd plasmids

The full-length human ULK1 with FLAG tag in C-terminal and EcoRI/SpeI sites in both ends was generated by PCR amplification. The human ULK1 cDNA (GenBank, NM_003565.2) was used as a template, and ‘primer 4’, ‘primer 5’ and ‘primer 6’ were designed to introduce FLAG tag and EcoRI/SpeI sites. Similarly, the ATG16L1 cDNA (GenBank NM_030803.7) was amplified and modified by introducing EcoRI/SpeI sites through PCR using hATG16L1-Flag as a template and the ‘primer 7’, ‘primer 8’ ‘primer 9’ as primers. The modified ULK1 or ATG16L1 DNA and pLVX-ZsGreen-Bsd plasmid (Supplementary Fig. S2a) were digested with restriction enzymes EcoRI and SpeI and ligated by T4 DNA ligase (New England Biolabs, Beijing, China) (Supplementary Fig. S2b, c) to develop pLVX-hULK1/-hATG16L1-3flag-ZsGreen-Bsd plasmids.

### Construction of pLVX-shRNA2-Bsd-hATG16L1 plasmid

Human ATG16L1 mRNA (GenBank: NM_030803.7) was employed as the template strand, and an online shRNA design tool was used to obtain the target gene interference sequence (http://rnaidesigner.thermofisher.com/rnaiexpress/sort.do). In this study, we designed three ATG16L1 target sequences to construct the lentiviral shRNAs. ATG16L1 target sequence fragments were synthesized by PCR using the ‘primer 10’, ‘primer 11’, and ‘primer 12’, with BamHI/EcoRI cleavage sites and FLAG tag inserted. Oligonucleotides were annealed, digested, and then inserted between the BamHI and EcoRI restriction sites of the plasmid vector pLVX-shRNA2-Bsd (Supplementary Fig. S2d) to develop pLVX-shRNA2-Bsd-hATG16L1 plasmid.

### Establishment of stable R778L HepG2 cell line with hULK1 overexpression, hATG16L1 overexpression, or hATG16L1 knockdown

The lentivirus expression plasmids pLVX-hULK1-3flag-ZsGreen-Bsd/pLVX- hATG16L1-3flag-ZsGreen-Bsd/LVX-shRNA2-Bsd-hATG16L1 were transfected with the packaging plasmids into HEK 293T cells for lentivirus generation. Lentiviruses were harvested at 24 and 48 h post-infection, centrifuged to remove cell debris, and filtered through 0.45 μm cellulose acetate filters. The ATP7B R778L HepG2 cells were subcultured at 5 × 10^5^ cells per well into six-well tissue culture plates. After 24 h culture, cells were infected with recombinant lentivirus at a multiplicity of infection (MOI) of 50.

### Cell viability assay

HepG2 cells were seeded into a 96-well plate. After treatment, the medium was carefully discarded, and 10 μL of Cell Counting Kit-8 (CCK-8; Dojindo, Kumamoto, Japan) was subsequently added to each well and incubated at 37 °C for 20 min. Finally, the absorbance was measured at 450 nm using a microplate spectrophotometer (Bio-Rad), and the cell viability was calculated.

### RNA isolation and RT-qPCR

Total RNA was extracted from cultured cells using TRIzol reagent (Invitrogen, Carlsbad, CA, USA). Then, 1 μg RNA was reverse transcribed into cDNA using the AMV retrotranscriptase system (TaKaRa, Dalian, Liaoning, China). qPCR reactions were run in triplicate on an ABI StepOne Plus System (Thermo Fisher Scientific) using SYBR Green reaction mix (TaKaRa, Dalian, Liaoning, China). The primers were designed by Primer Version 0.4.0 and listed in Supplementary Table 1. The relative expression of the target gene was calculated and normalized to the expression of the reference gene *Gapdh*.

### Western blot

Whole proteins from cells were extracted using RIPA solution containing protease and phosphatase inhibitor cocktail (Thermo Fisher Scientific). Protein concentrations were assessed with the bicinchoninic acid (BCA) Protein Quantification kit. Proteins were then subjected to 10% SDS-PAGE and transferred onto polyvinylidene difluoride (PVDF) membranes (Thermo Fisher Scientific). The membranes were blocked with 5% skim milk for 1 h at room temperature and then probed with the following primary antibodies overnight at 4 °C: RIP1(E8S7U) XP rabbit mAb (1:1000; Cell Signaling Technology), RIP3 (E7A7F) XP rabbit mAb (1:1000; Cell Signaling Technology), Phospho-RIP3 Rabbit mAb (1:1000; Cell Signaling Technology), Anti-MLKL antibody (1:2000; Abcam), phospho-MLKL (Ser345) (D6E3G) rabbit mAb (1:1000; Cell Signaling Technology), Atg16L1 (D6D5) Rabbit mAb (1:1000; Cell Signaling Technology), Phospho-Atg16L1 (Ser278) (E7K6H) Rabbit mAb (1:1000; Cell Signaling Technology), ULK1 (D8H5) Rabbit mAb (1:1000; Cell Signaling Technology), Phospho-ULK1 (Ser757) (D7O6U) Rabbit mAb (1:1000; Cell Signaling Technology), and LC3B (D11) Rabbit mAb (1:1000; Cell Signaling Technology). After washing, the membranes were incubated with HRP-conjugated anti-rabbit IgG for 1 h at room temperature. The protein bands were visualized by Luminol ECL reagent (Thermo Fisher Scientific). β-actin, β-tubulin, and GAPDH were used as endogenous controls.

### Differentially expressed gene (DEG) analysis

The GSE125637 (GPL1261) dataset for Atp7b-/- mouse, whose hallmarks are similar to Wilson’s disease, is downloaded from the Gene Expression Omnibus (GEO) database (http://www.ncbi.nlm.nih.gov/geo/). The autophagy-related genes included LC3II, ULK1, ATG16L1, RIPK1, RIPK3, and MLKL.

The Network-Analyst online tool (https://www.networkanalyst.ca/) and limma R package (version 3.44.3) were used to analyze differentially expressed genes (DEGs) between different groups. The adjustive *P* < 0.05 and |Log2 fold change (logFC)| >1 were set as the parameters to identify DEGs. The Wilcoxon test was employed to compare the statistical differences between the two groups.

### Statistical analysis

The results were expressed as mean ± standard error of the mean or median (Min, Max). Group comparisons were performed using Student’s *t-*test, Mann–Whitney U test, or one-way ANOVA followed by Tukey’s multiple comparison test, as appropriate. Statistics and graphs were generated using Prism 6.0 software (GraphPad Software Inc., San Diego, CA, USA). *P* < 0.05 was considered statistically significant.

### Supplementary information

Supplementary Figure

WB

Supplementary Table

## Data Availability

The dataset generated during the current study is available from the corresponding author upon reasonable request.
