# Supplementary material for: ATP7B R778L mutant hepatocytes resist copper toxicity by activating autophagy and inhibiting necroptosis
Source: Cell Death Discov. 2023 Sep 16;9:344. doi: 10.1038/s41420-023-01641-5 (PMC10505209; doi:10.1038/s41420-023-01641-5)
Supplement: Supplementary file 1 — Supplementary Figure [file 41420_2023_1641_MOESM1_ESM.pdf]

Figure S1

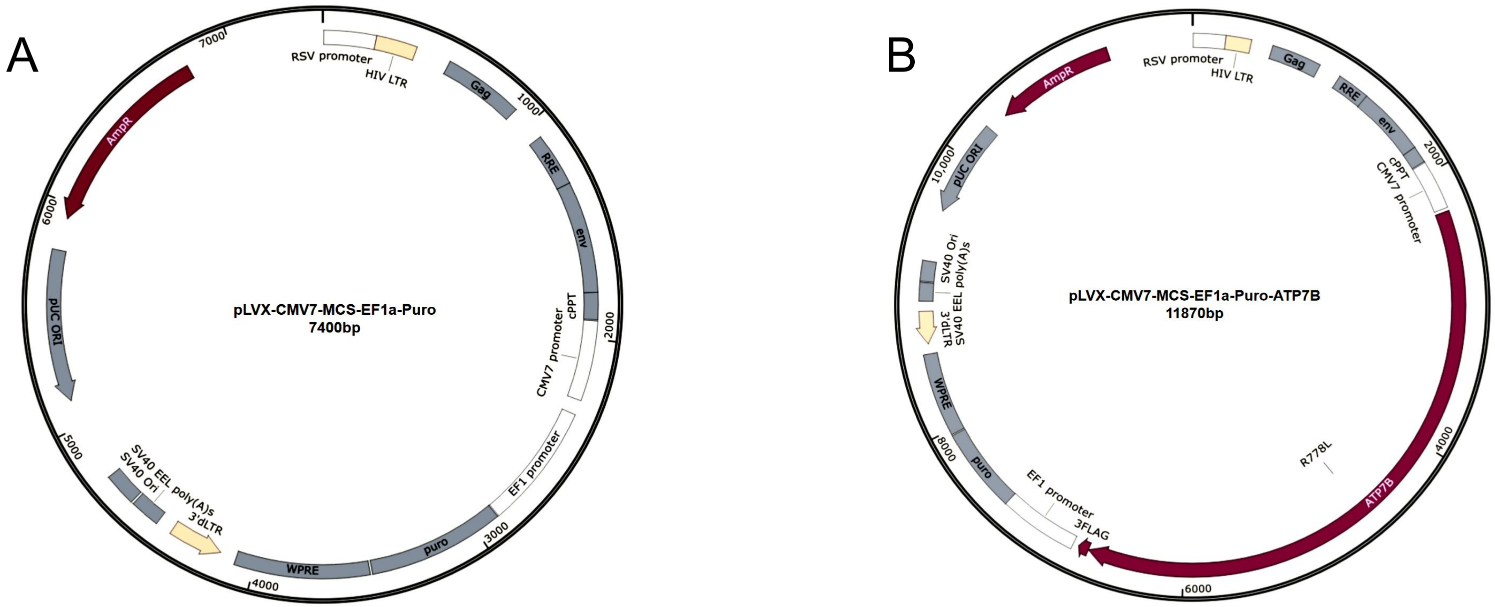

Figure S1. The lentiviral vector used to constructed ATP7B R778L overexpression model. A. The blank lentiviral vector pLVX-CMV7-MCS-EF1a-Puro. B. The final lentiviral vector with ATP7B R778L and FLAG tag cloned.

Figure S2

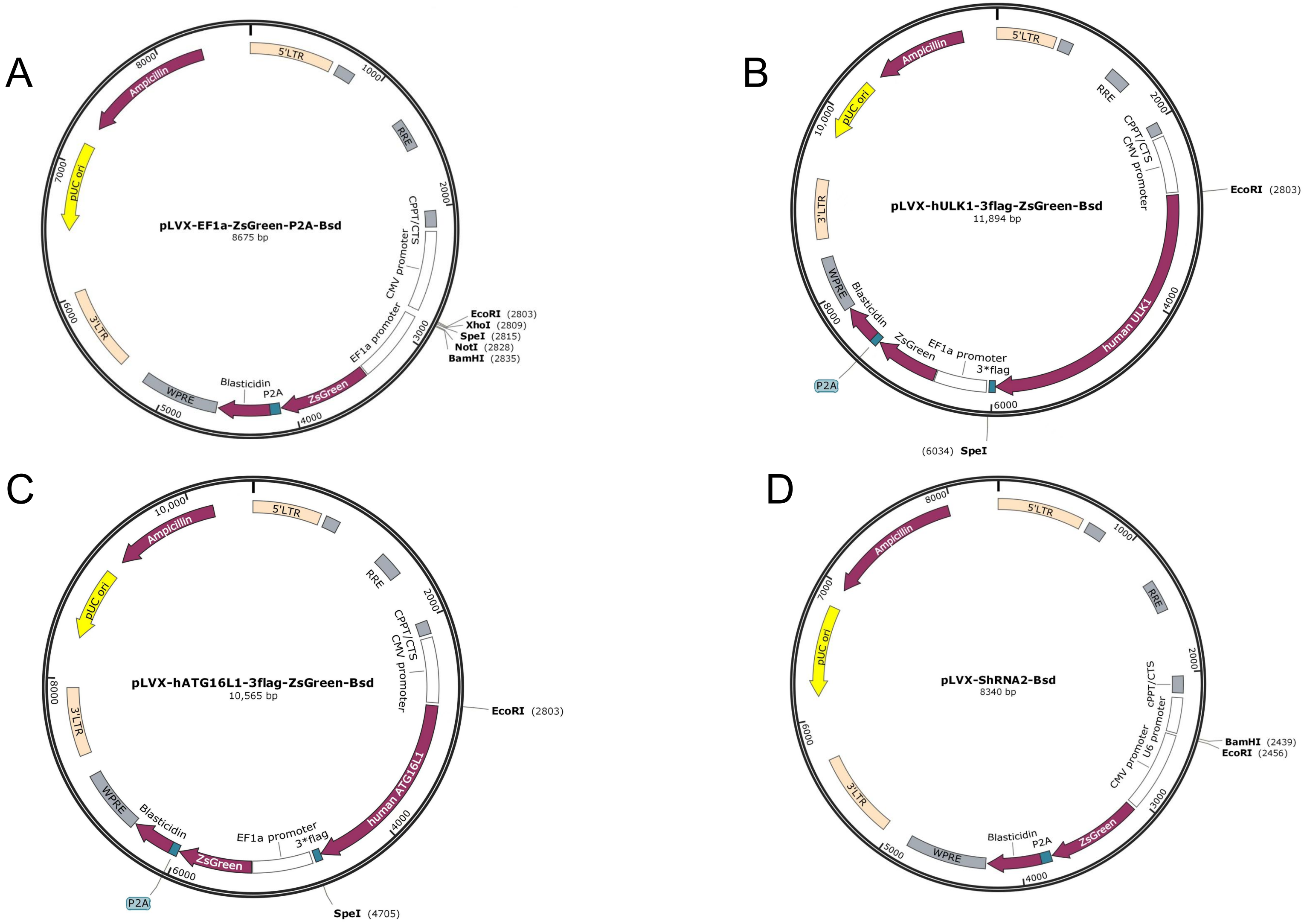

Figure S2. The plasmid vector used in this research. A. The blank plasmid vector pLVX-ZsGreen-Bsd plasmid. B. The plasmid vector with ULK1 overexpression. C. The plasmid vector with ATG16L1 overexpression. D. The plasmid vector pLVX-shRNA2-Bsd with ATG16L1 knockdown.

Figure S3

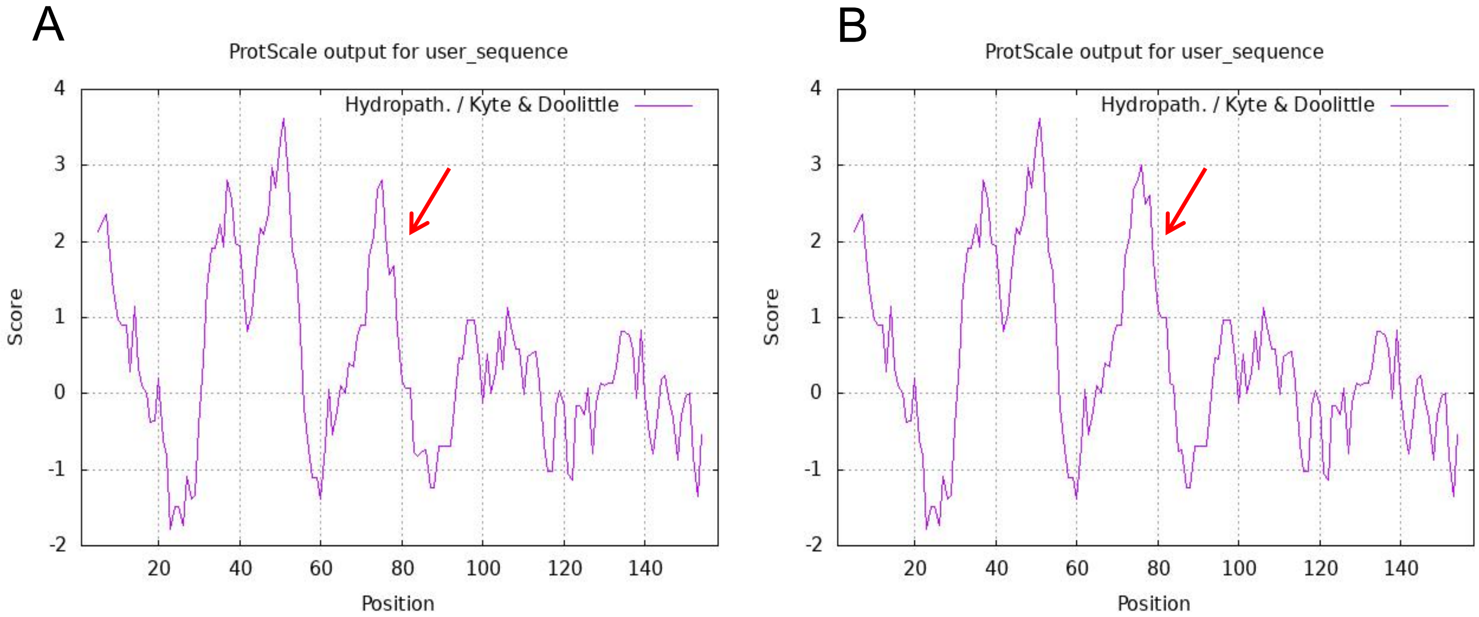

Figure S3. Hydrophobicity analysis results. A. The hydrophobicity of wild type ATP7B protein (Red arrow, hydrophilic amino acid Arg). B. The hydrophobicity of ATP7B R778L protein (Red arrow, hydrophobic amino acid Leu).

Figure S4

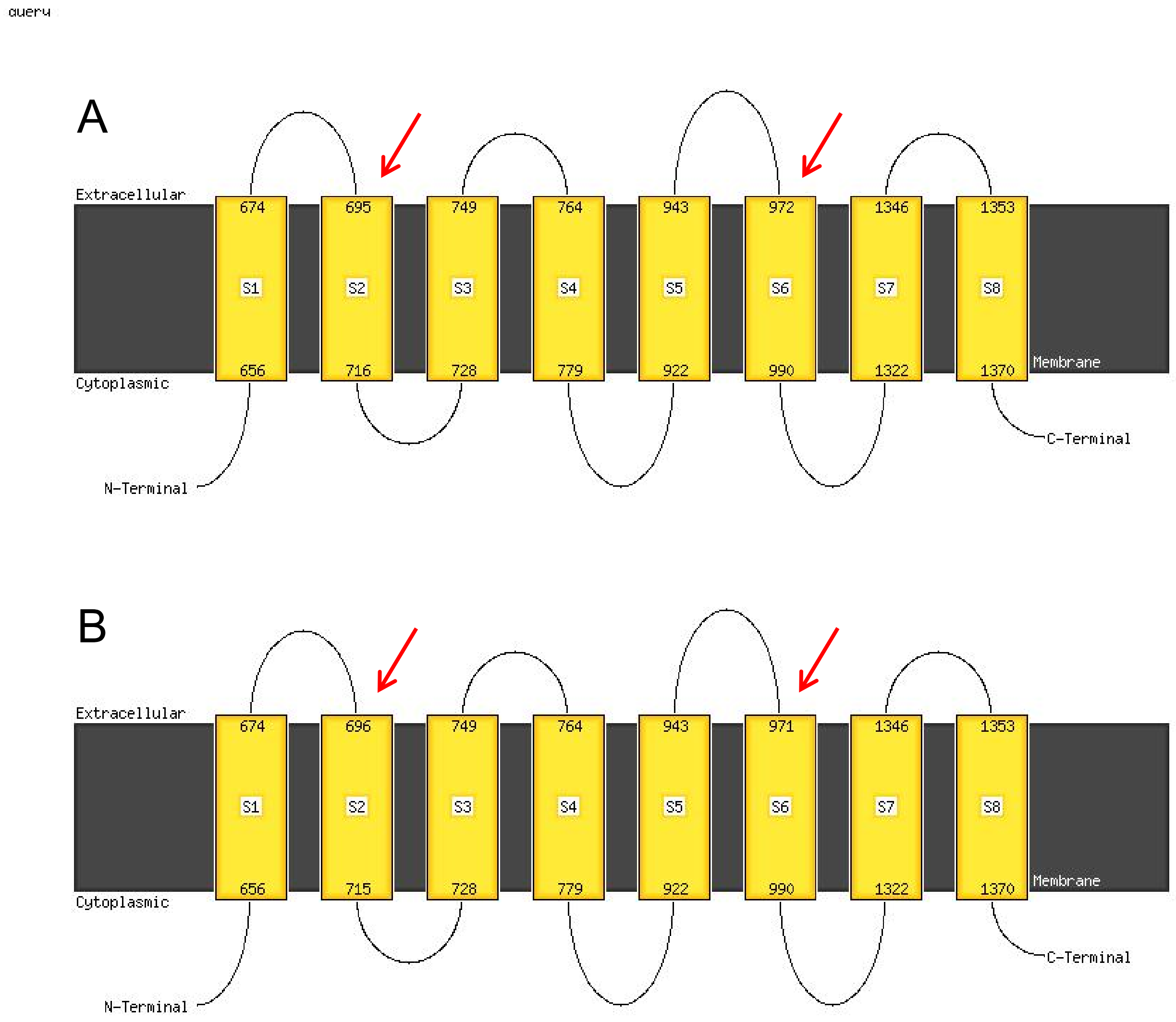

Figure S4. The transmembrane structure. A. The transmembrane structure of ATP7B WT protein. B. The transmembrane structure of ATP7B R778L protein (Red arrow, the S2 and S6 transmembrane helix were changed).

Figure S5

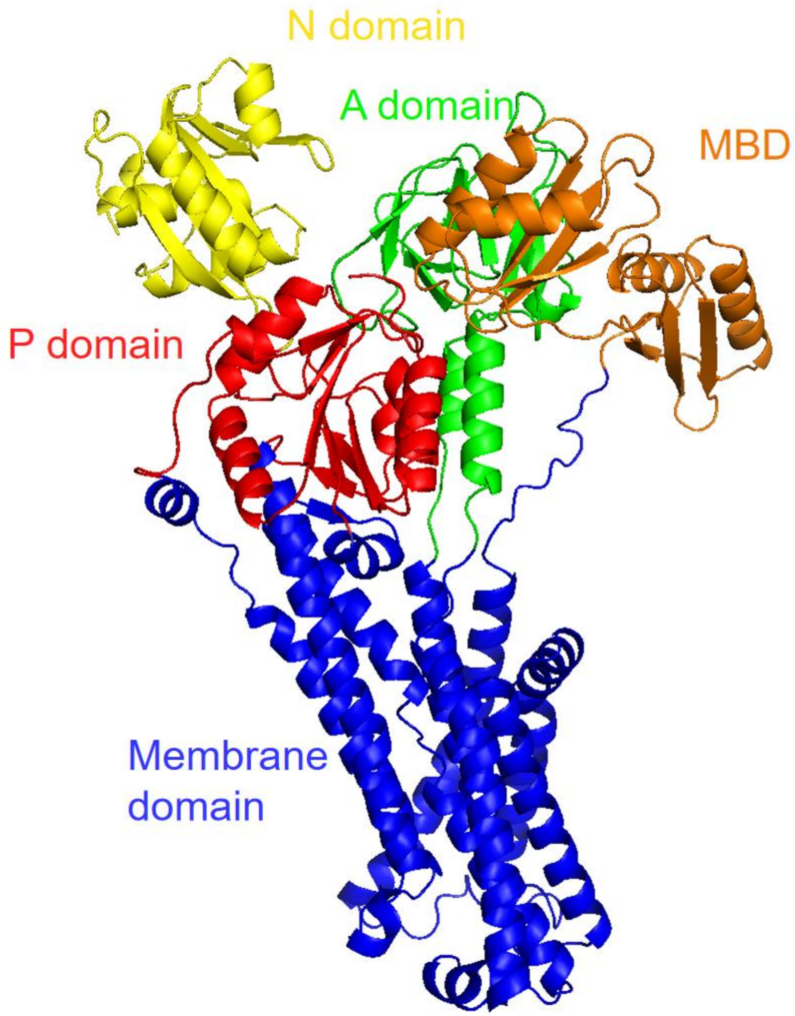

Figure S5. 3D structure of ATP7B protein.

Figure S6

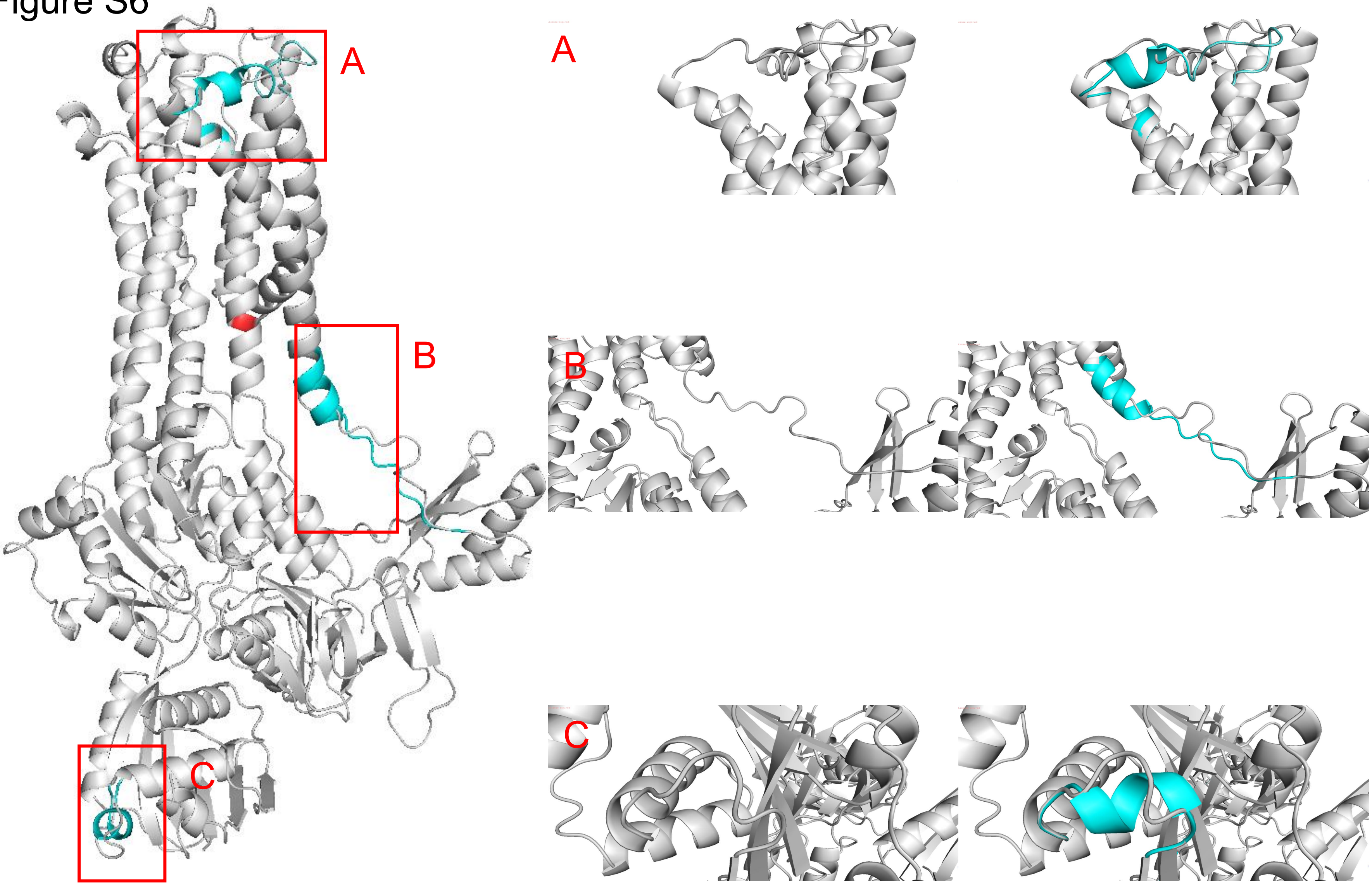

Figure S6. The superposition of the R778L mutant ATP7B protein with the wild-type ATP7B three-dimensional structure.

Figure S7

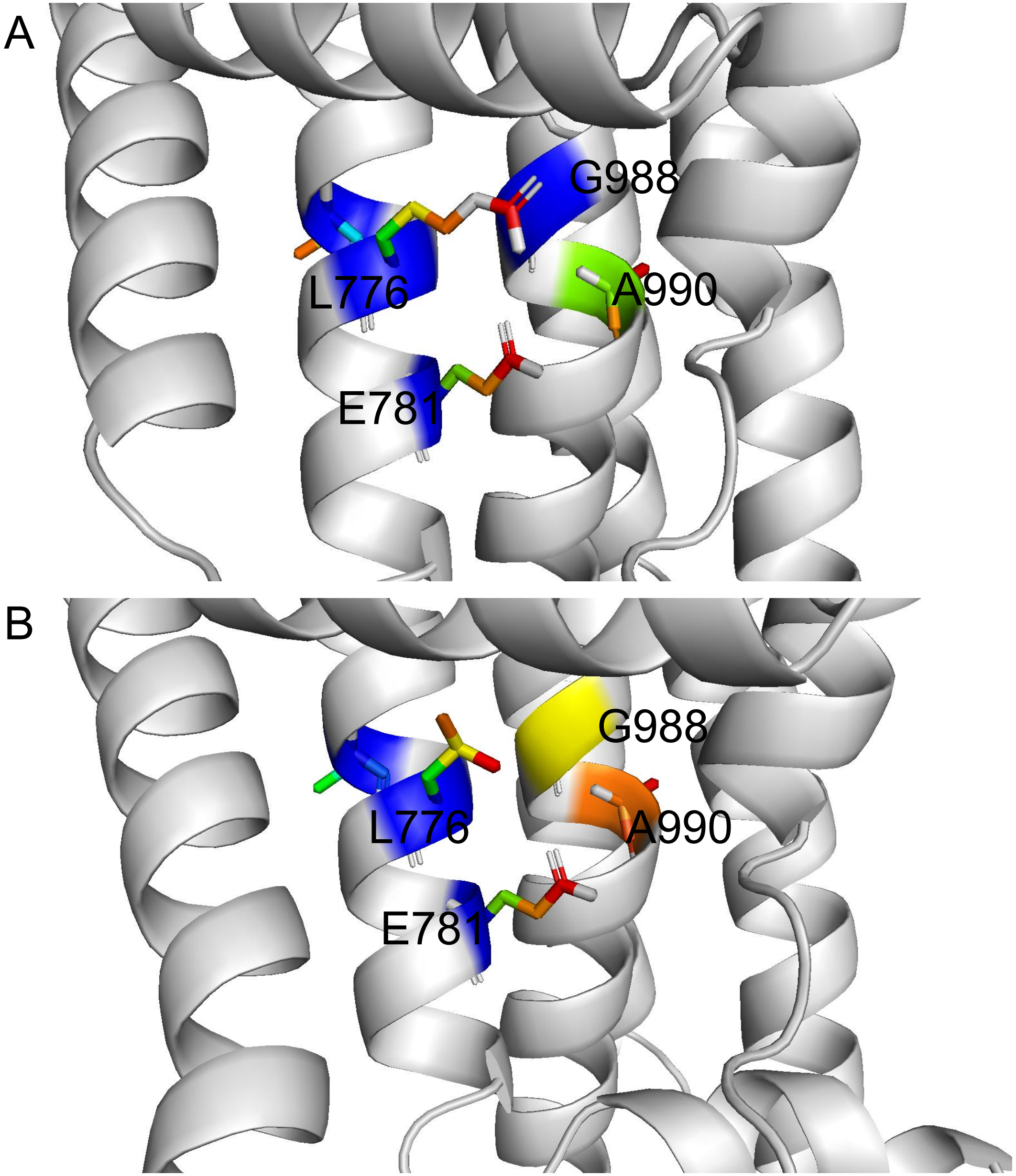

Figure S7. The effects of the ATP7B R778L mutation on chemical bonding.

Figure S8

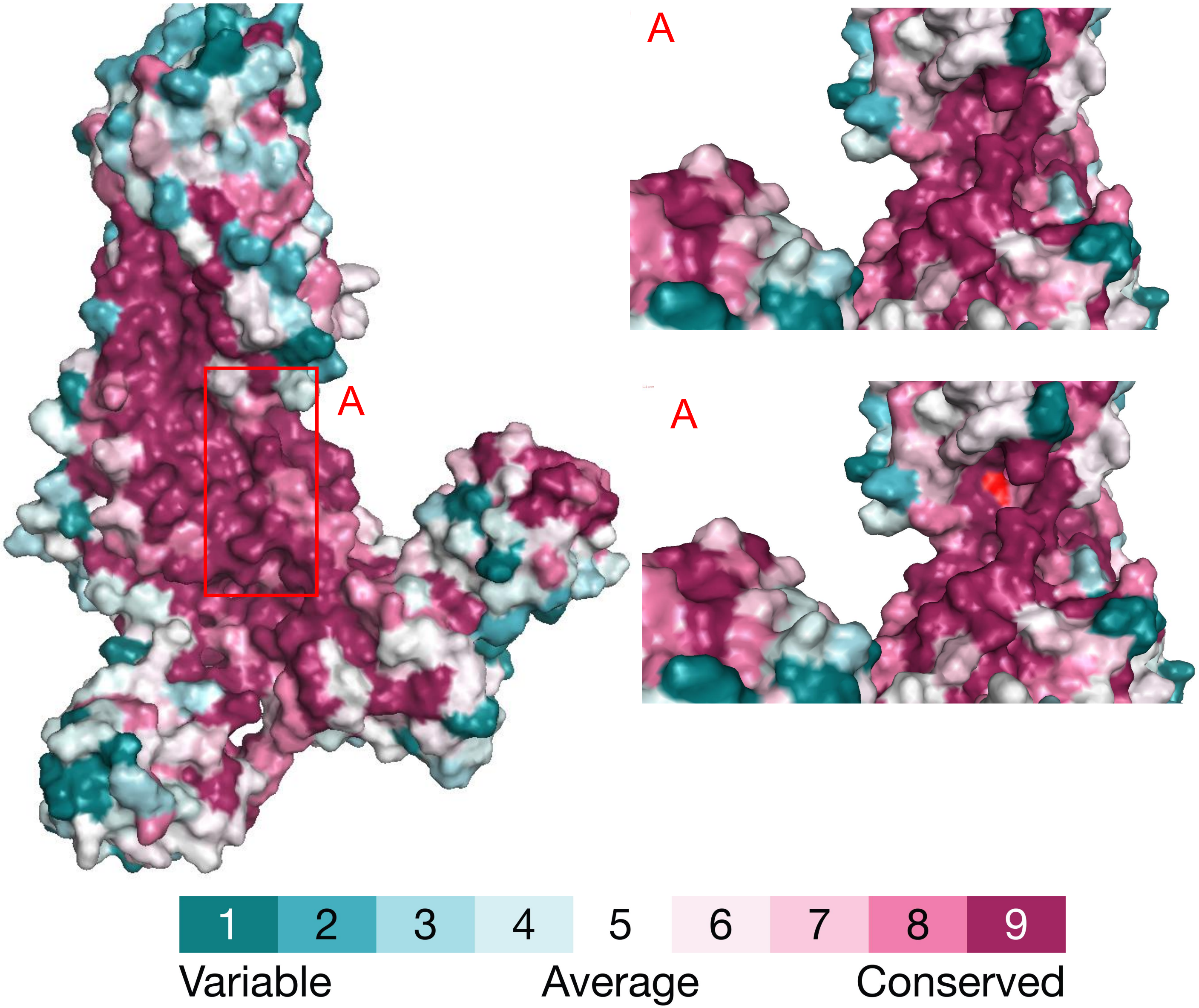

Figure S8. Conservation analysis of ATP7B protein. The red core showed that the 778 site was in the conservation area.

Figure S9

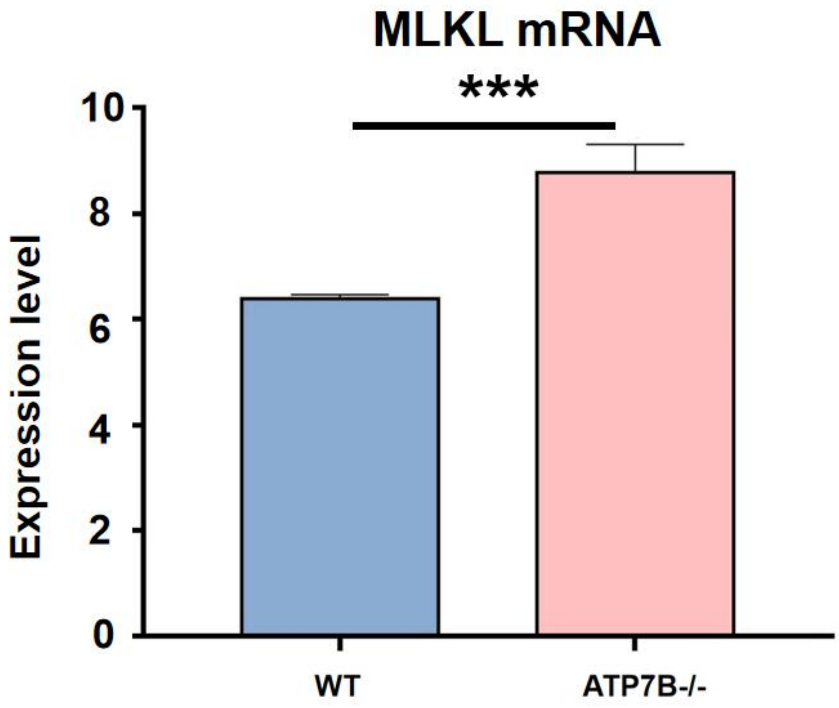

Figure S9. The expression levels of MLKL in the liver tissues of ATP7B-/- mice were significantly higher than those in the livers of wild-type controls through the gene data analysis.

Figure S10

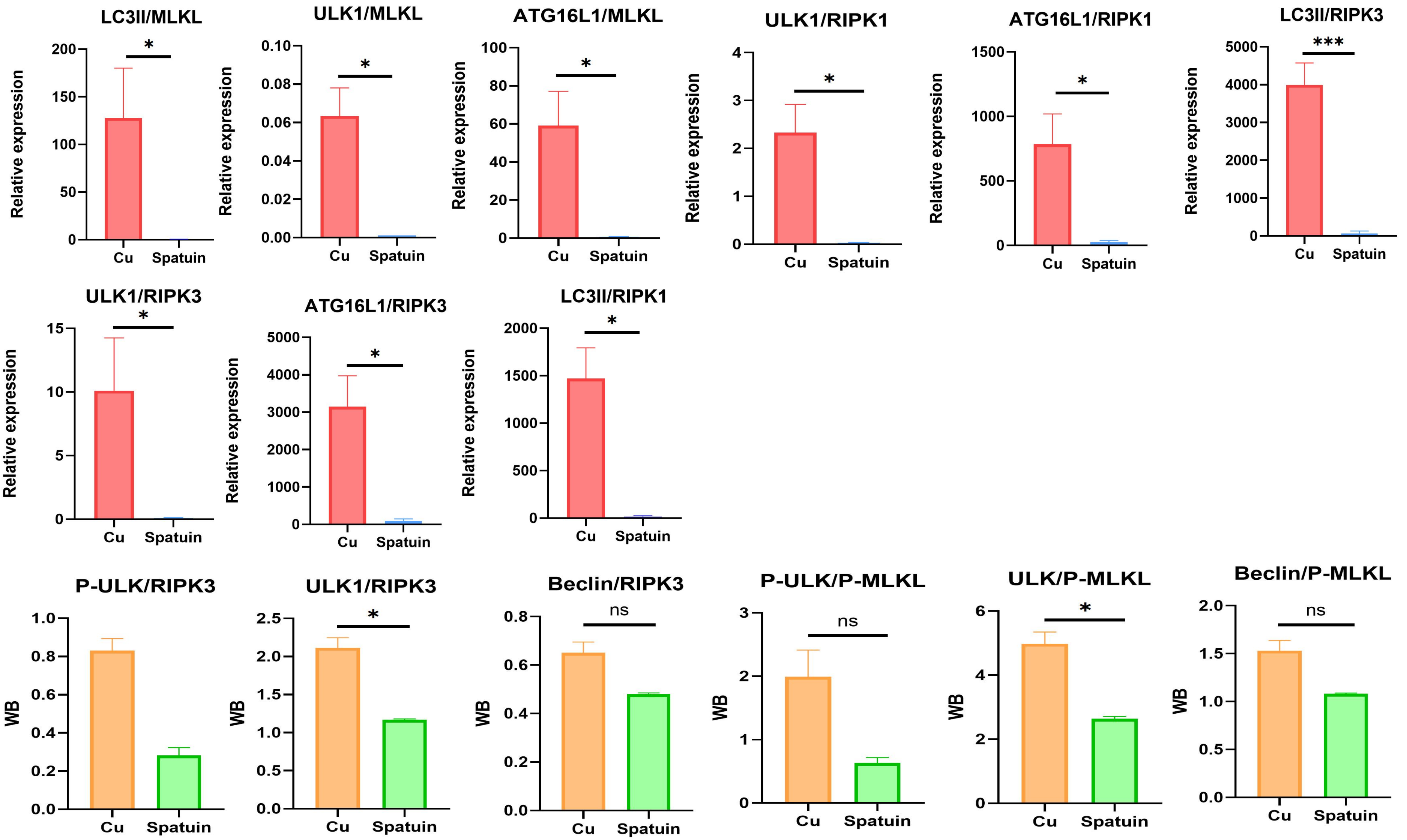

Figure S10. The ratios of autophagy to necroptosis in $CuSO_4$-treated cells with or without spautin-1 treatment at the mRNA and protein levels.

Figure S11

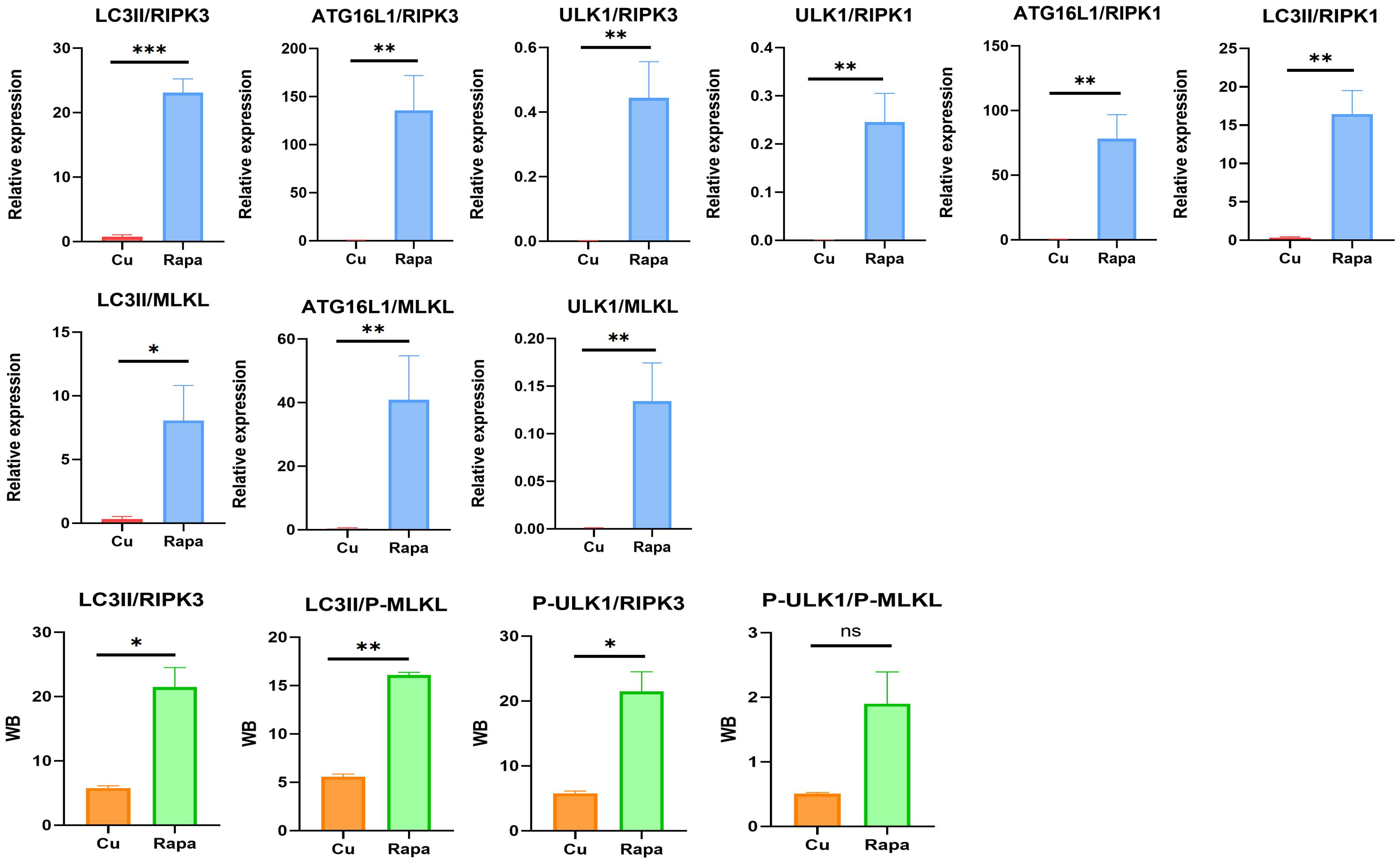

Figure S11. The ratios of autophagy to necroptosis in $CuSO_4$-treated cells with or without rapamycin treatment at the mRNA and protein levels.

Figure S12

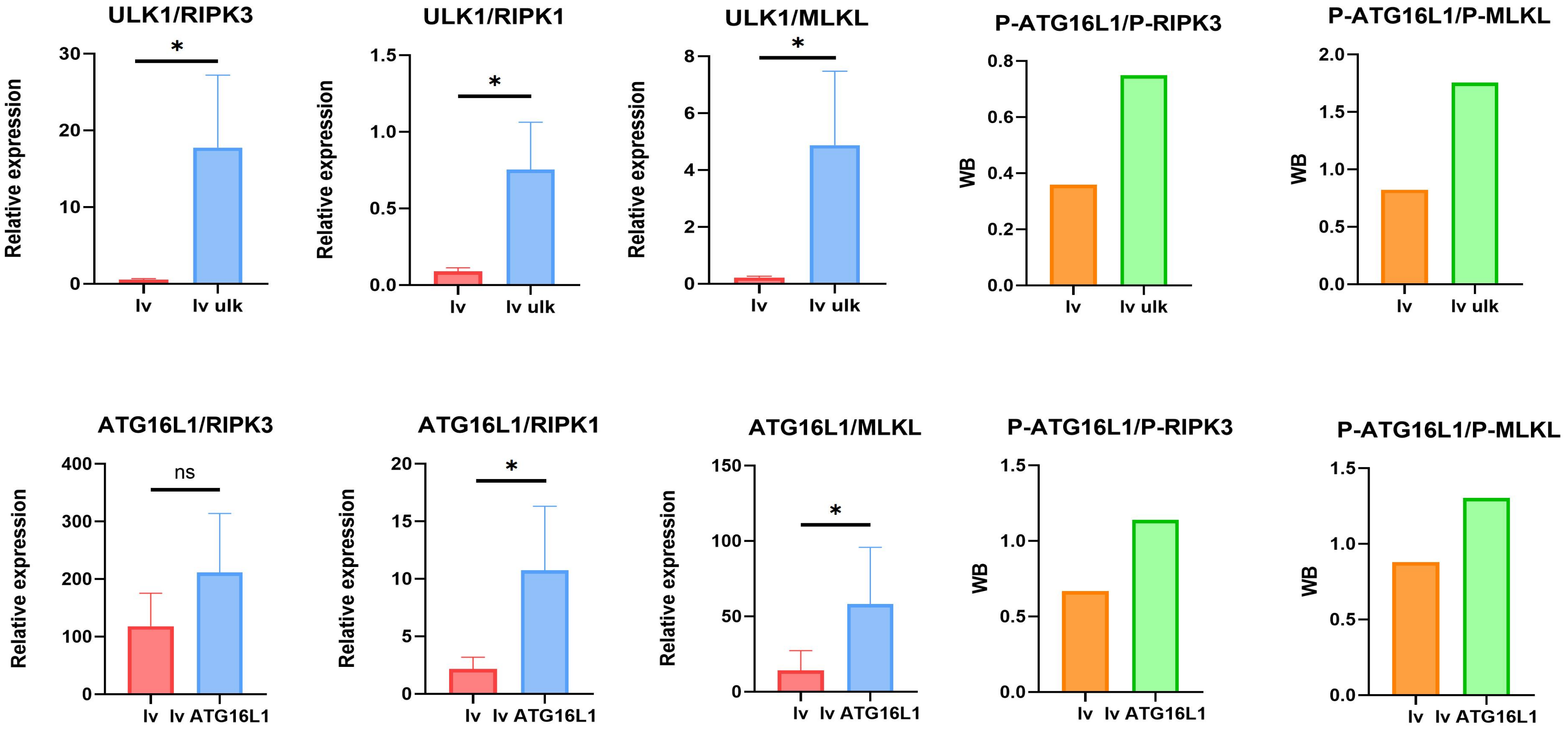

Figure S11. The ratios of autophagy to necroptosis in ULK1- or ATG16L1-overexpressed cells compared with control cells at the mRNA and protein levels.

Figure S13

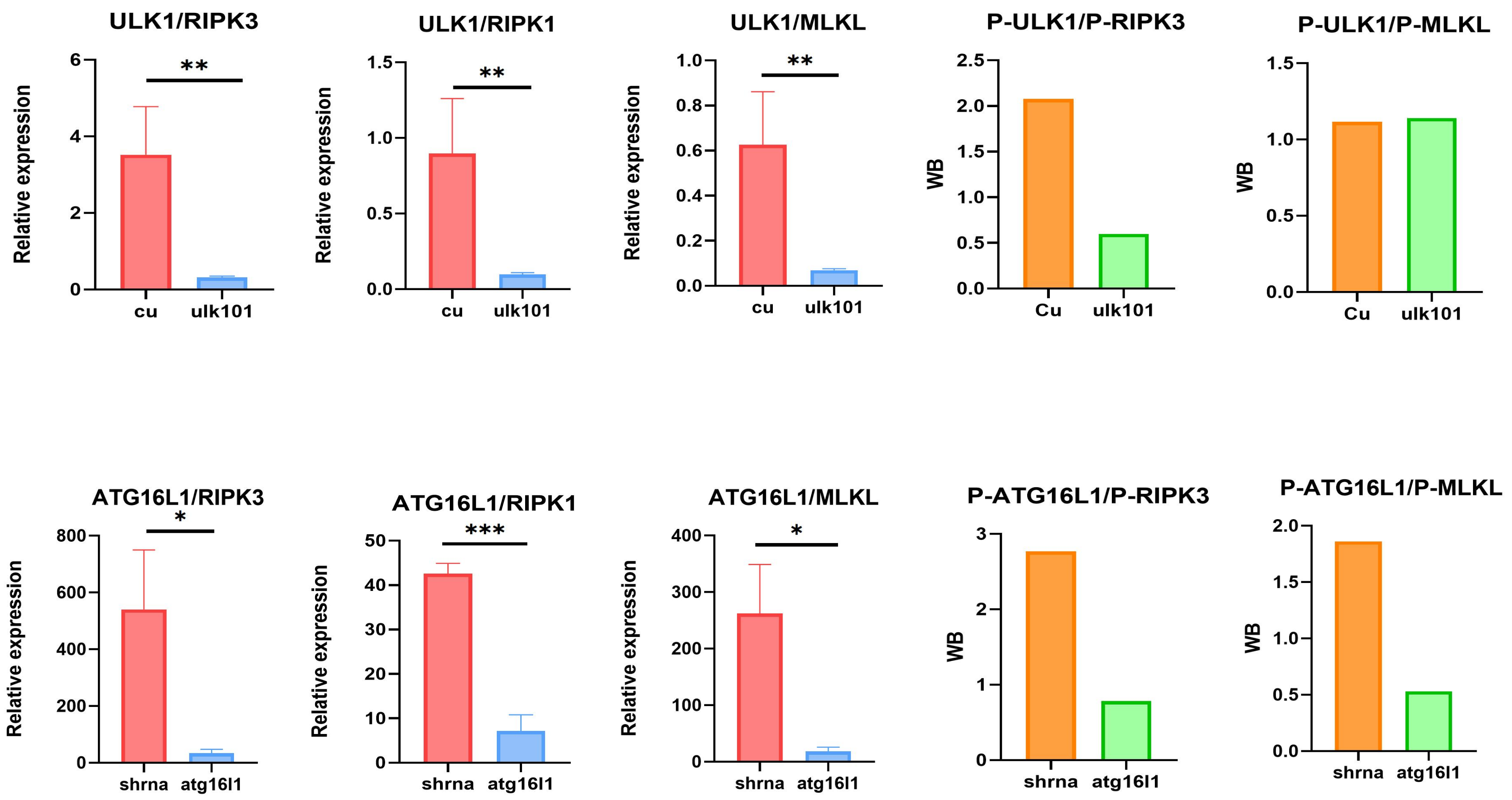

Figure S11. The ratios of autophagy to necroptosis in ULK1- or ATG16L1-knockdown cells compared with control cells at the mRNA and protein levels.
