# Supplementary material for: ATP7B R778L mutant hepatocytes resist copper toxicity by activating autophagy and inhibiting necroptosis
Source: Cell Death Discov. 2023 Sep 16;9:344. doi: 10.1038/s41420-023-01641-5 (PMC10505209; doi:10.1038/s41420-023-01641-5)
Supplement: Supplementary file 2 — WB [file 41420_2023_1641_MOESM2_ESM.pdf]

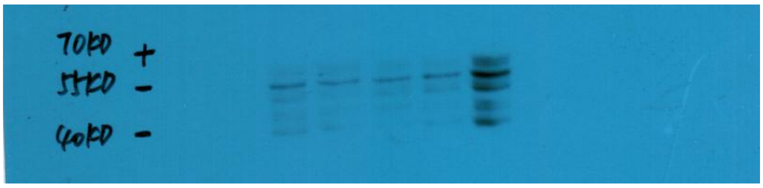

Figure 2B-1-P-MLKL

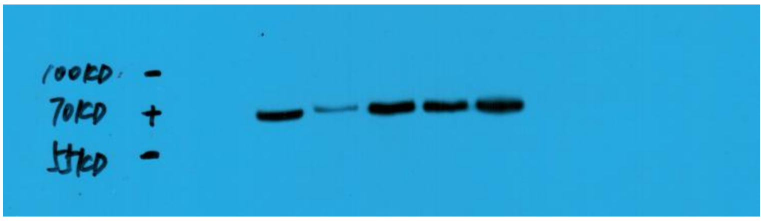

Figure 2B-2-RIPK1

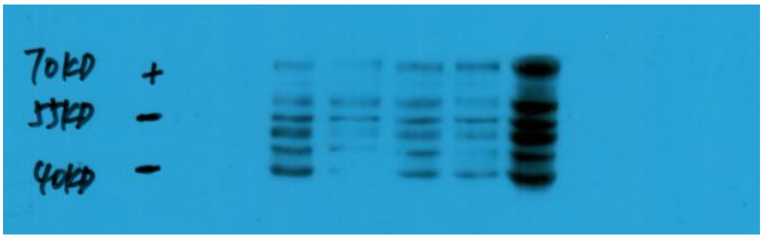

Figure 2B-3-P-RIPK3

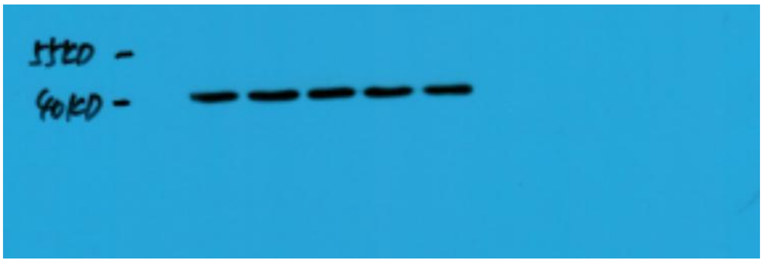

Figure 2B-4-beta actin

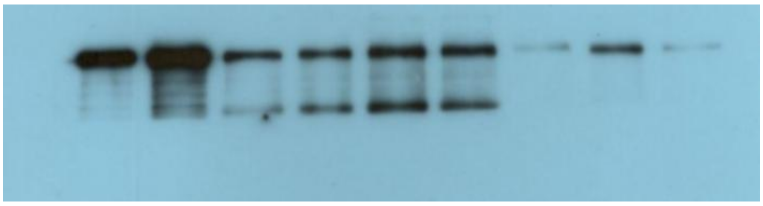

Figure 3B-1-R778L ULK1

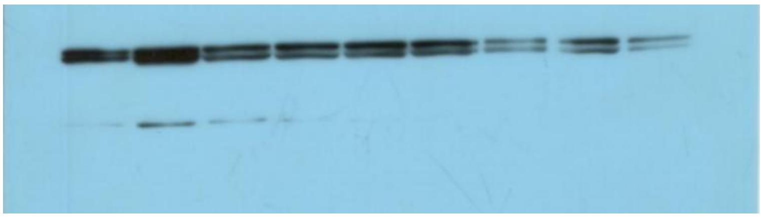

Figure 3B-2-R778L ATG16L1

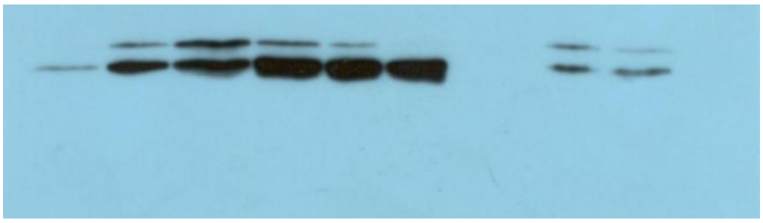

Figure 3B-3-R778L LC3B

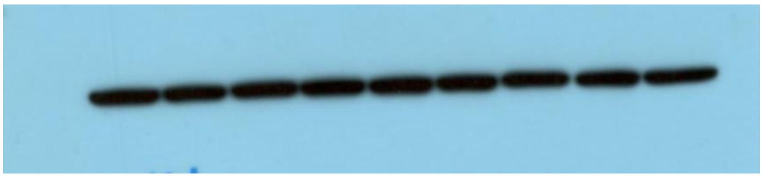

Figure 3B-4-R778L β tublin

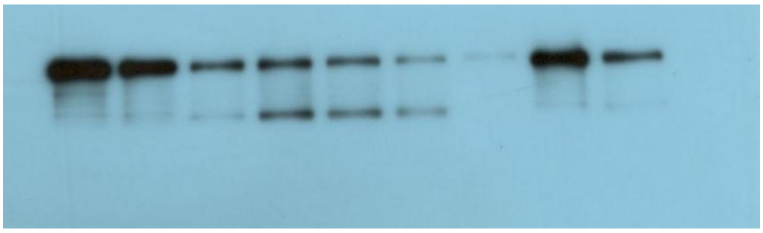

Figure 3B-5-WT ULK1

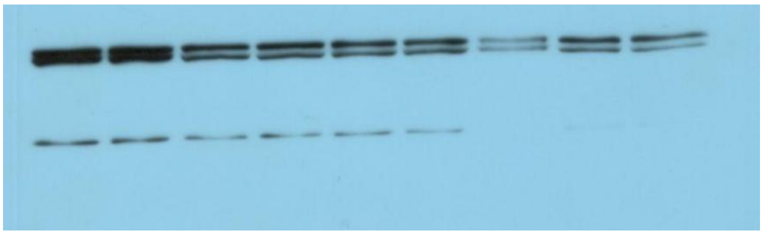

Figure 3B-6-WT ATG16L1

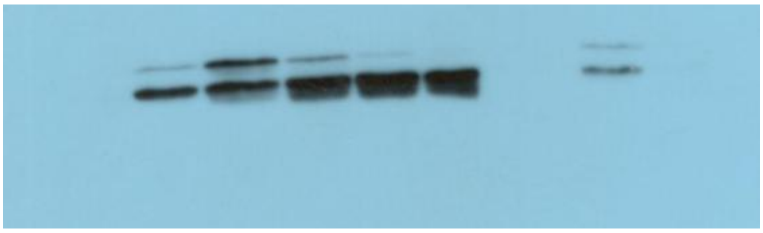

Figure 3B-7-WT LC3B

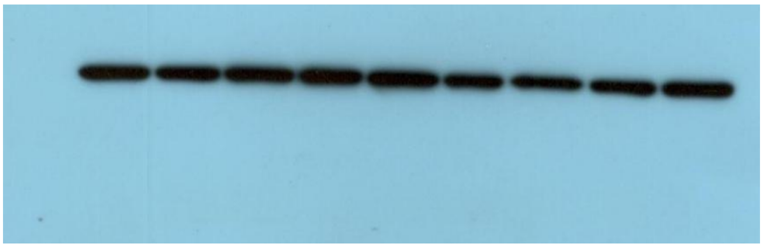

Figure 3B-8-WT β tublin

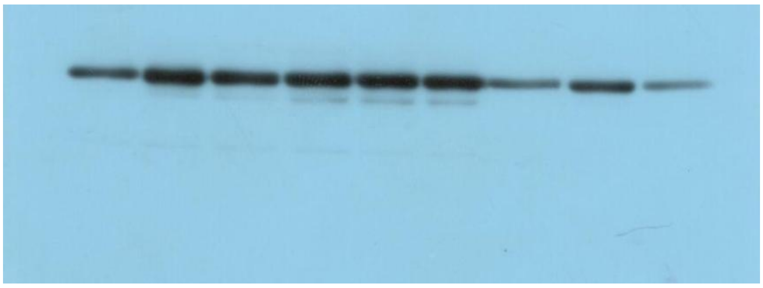

Figure 4B-1-R778L RIPK3

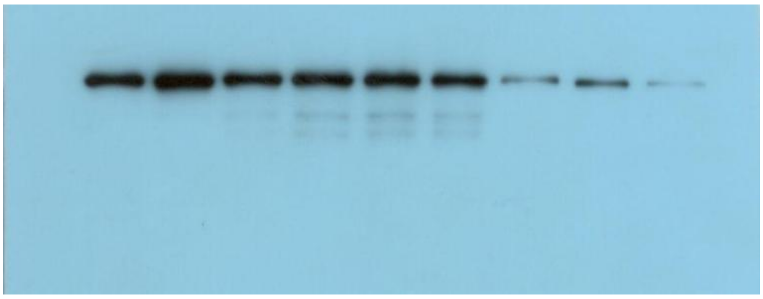

Figure 4B-2-R778L RIPK1

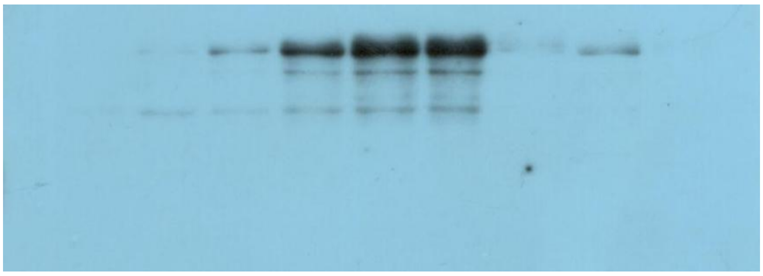

Figure 4B-3-R778L P-RIPK3

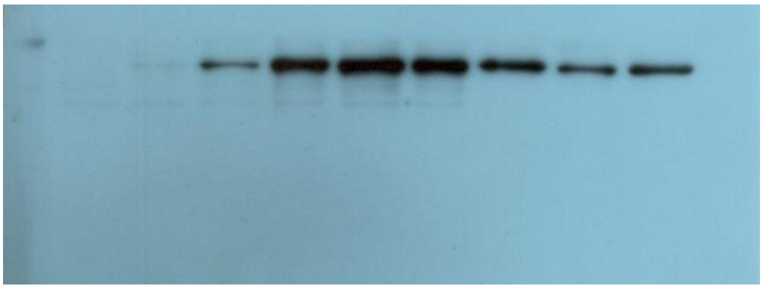

Figure 4B-4-R778L P-MLKL

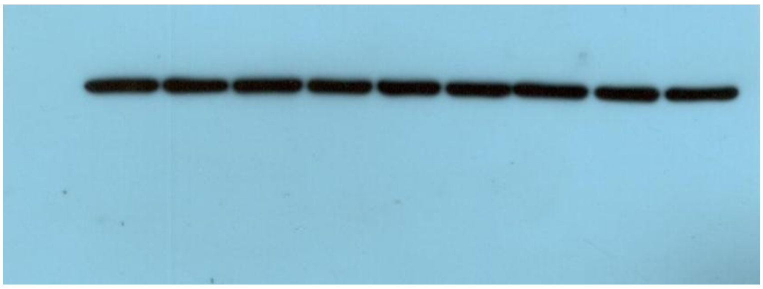

Figure 4B-5-R778L β tublin

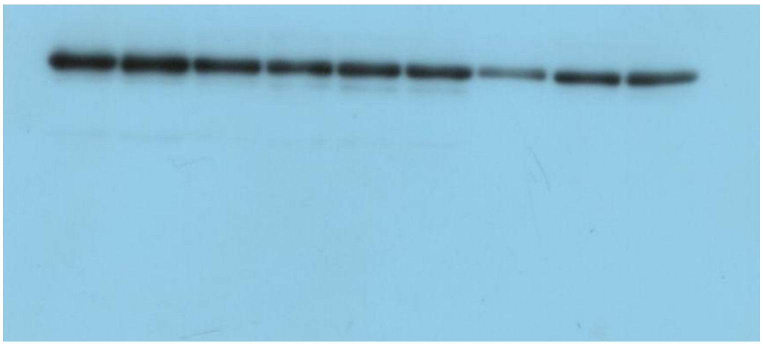

Figure 4B-6-WT RIPK3

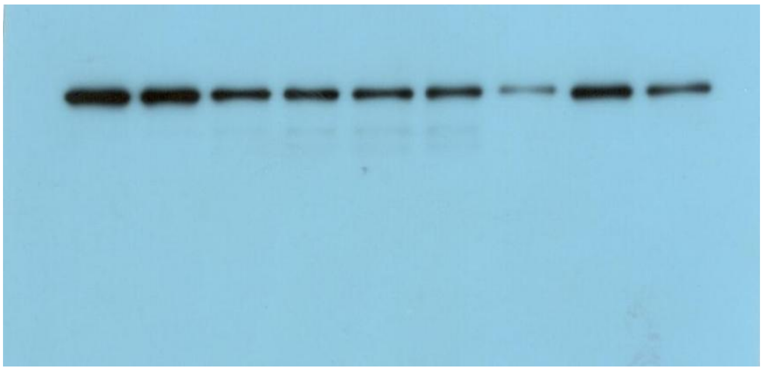

Figure 4B-7-WT RIPK1

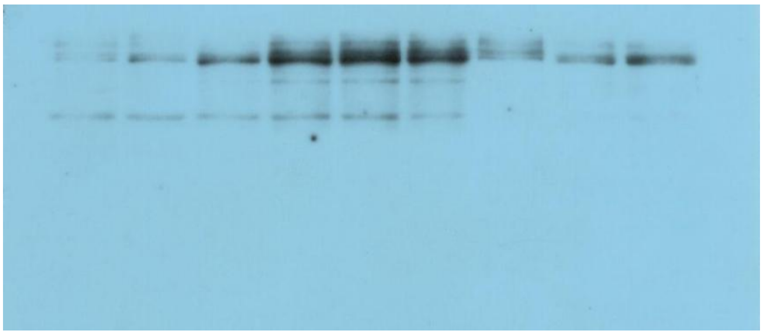

Figure 4B-8-WT P-RIPK3

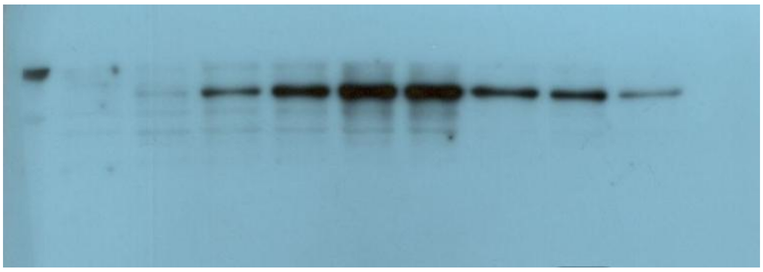

Figure 4B-9-WT P-MLKL

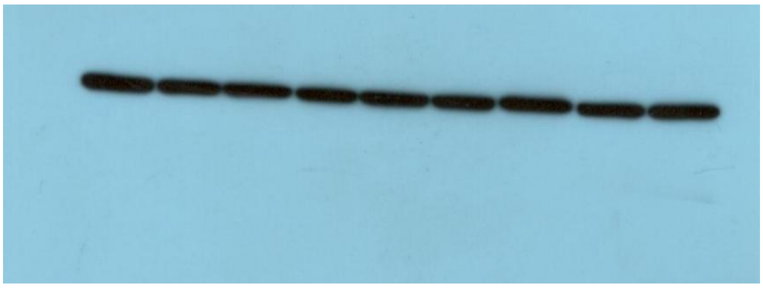

Figure 4B-10-WT β tublin

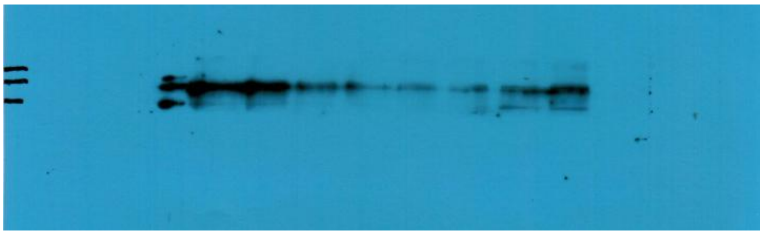

Figure 5B-1-P-ULK1

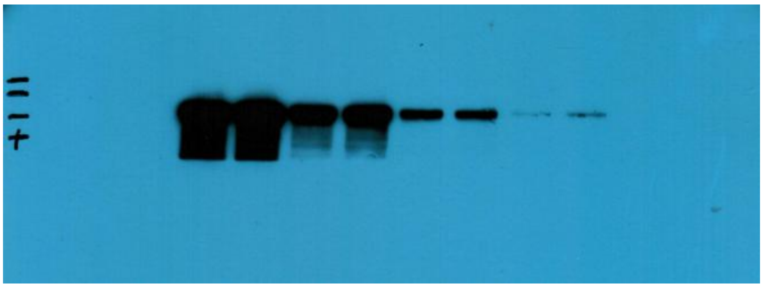

Figure 5B-2-ULK1

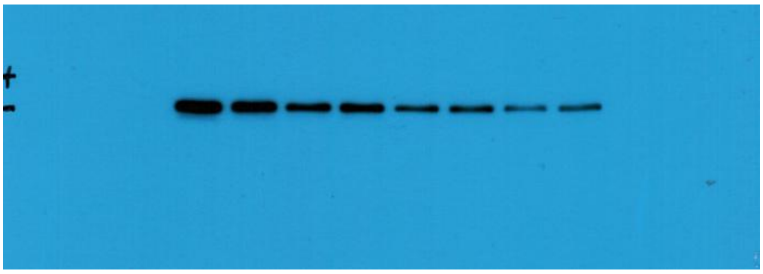

Figure 5B-3-Beclin1

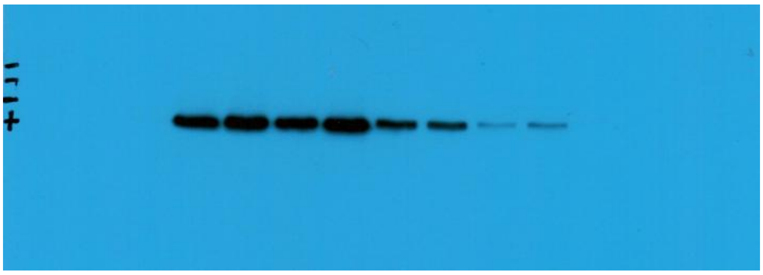

Figure 5B-4-RIPK3

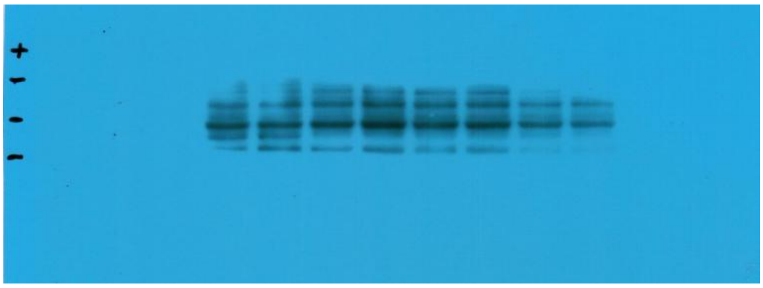

Figure 5B-5-P-MLKL

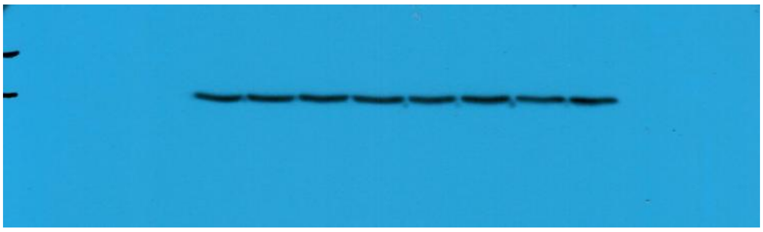

Figure 5B-6-GAPDH

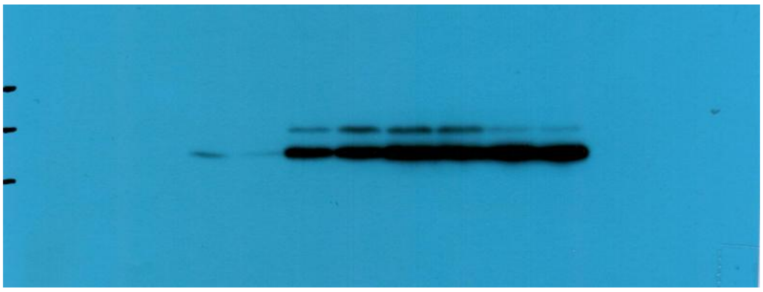

Figure 6B-1-LC3B

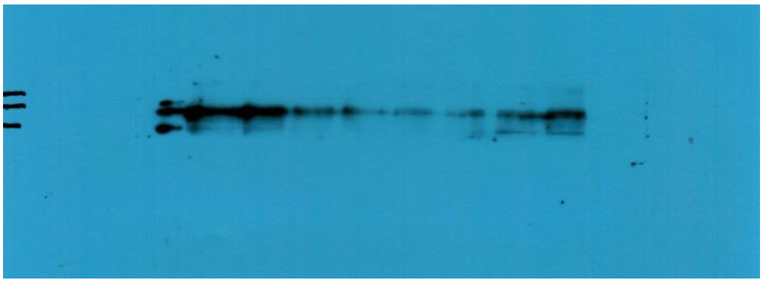

Figure 6B-2-P-ULK1

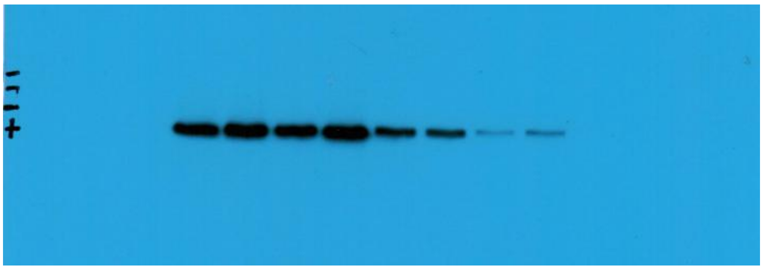

Figure 6B-3-RIPK3

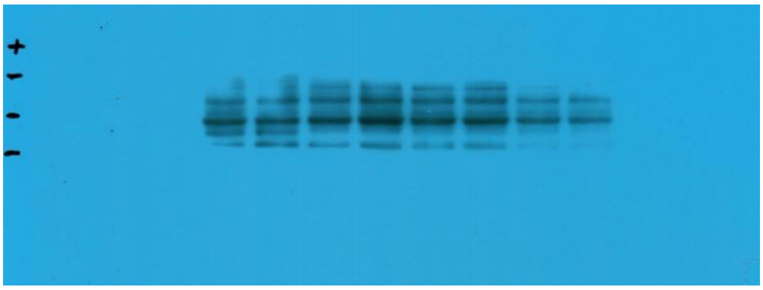

Figure 6B-4-P-MLKL

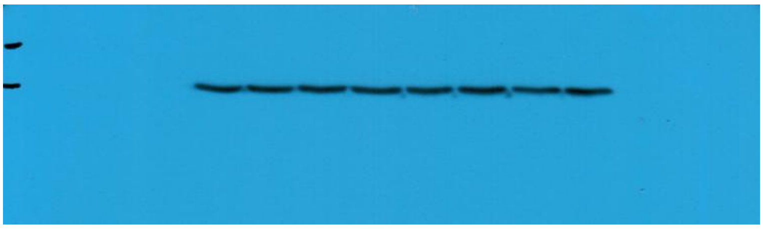

Figure 6B-5-GAPDH

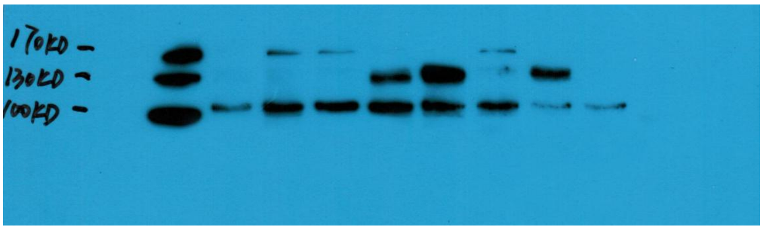

Figure 7C-1-P-ULK1

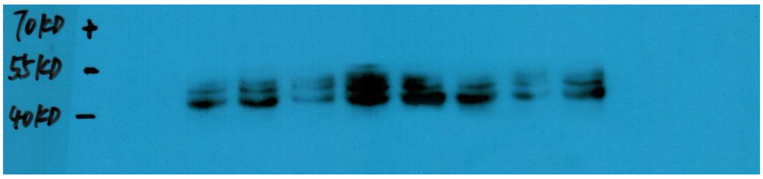

Figure 7C-2-P-RIPK3

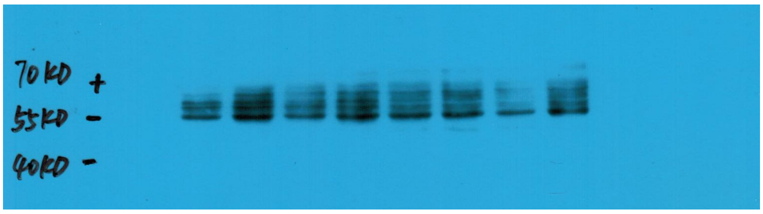

Figure 7C-3-P-MLKL

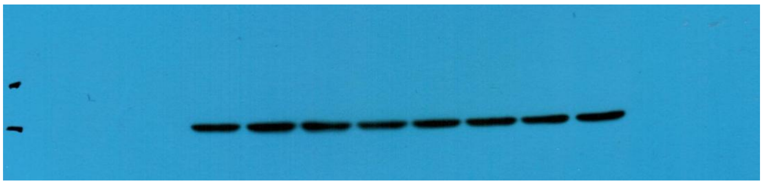

Figure 7C-4-β-actin

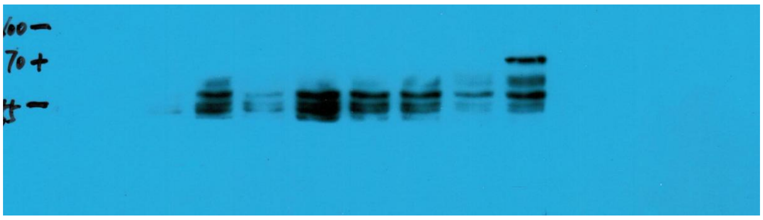

Figure 7D-1-P-ATG16L1

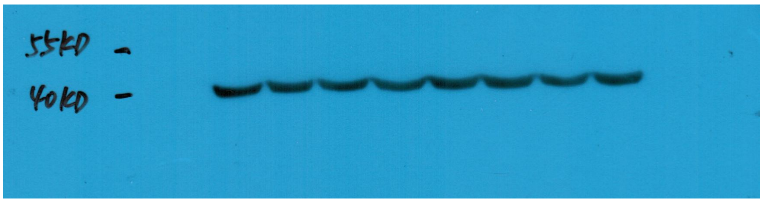

Figure 7D-2-β-actin

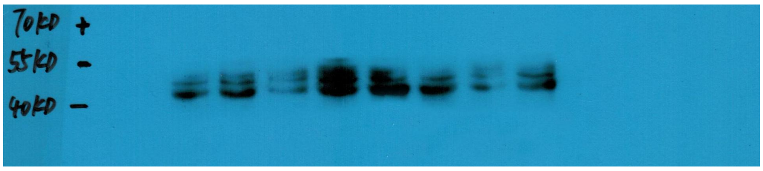

Figure 7D-3-P-RIPK3

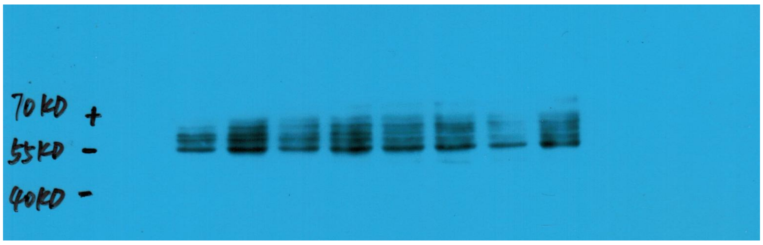

Figure 7D-4-P-MLKL

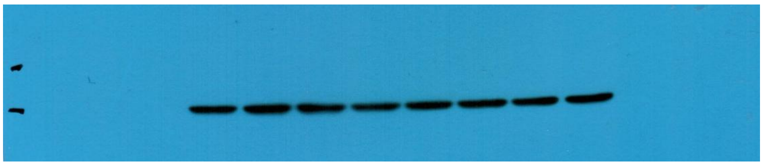

Figure 7D-5-β-actin

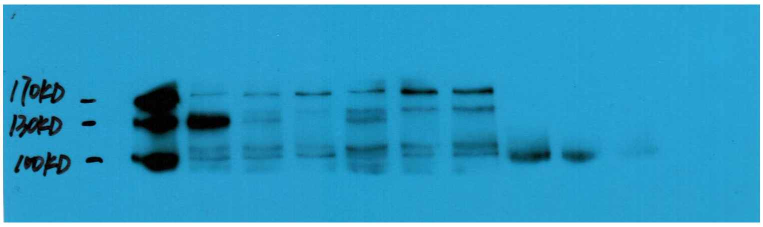

Figure 8C-1-P-ULK1

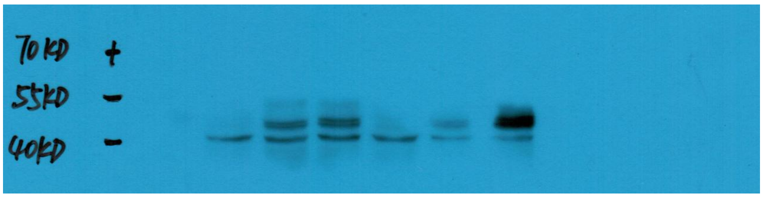

Figure 8C-2-P-RIPK3

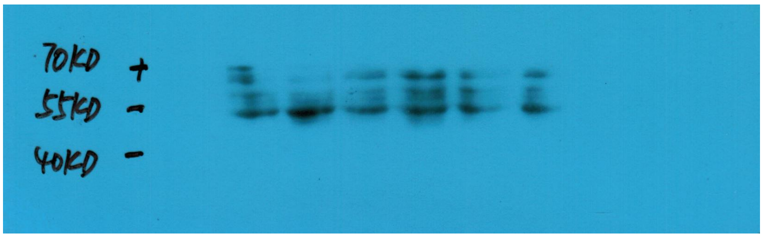

Figure 8C-3-P-MLKL

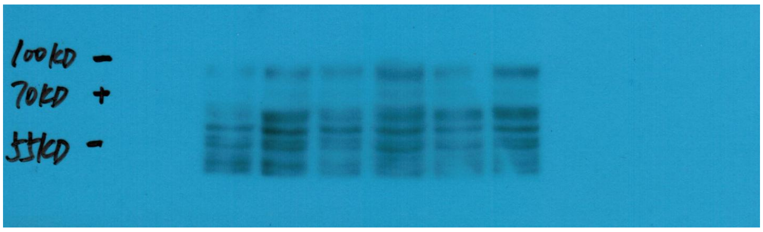

Figure 8F-1-P-ATG16L1

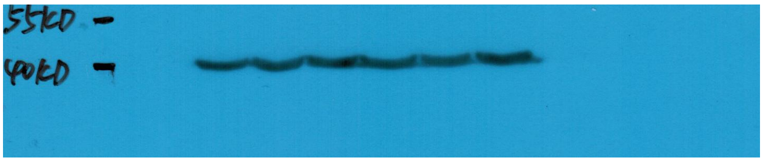

Figure 8F-2-β-actin

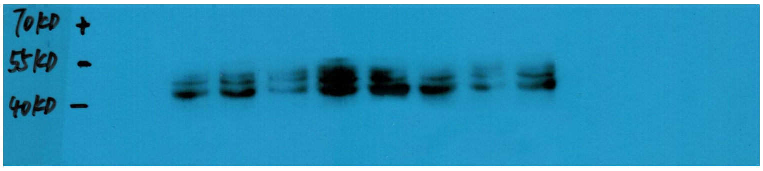

Figure 8F-3-P-RIPK3

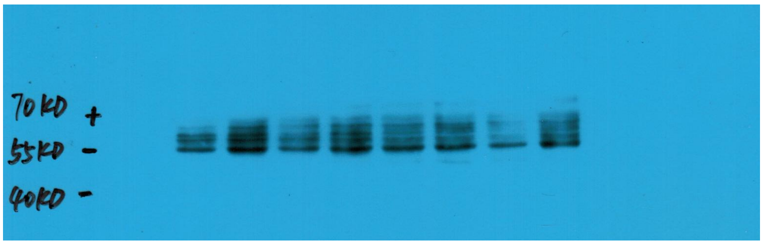

Figure 8F-4-P-MLKL

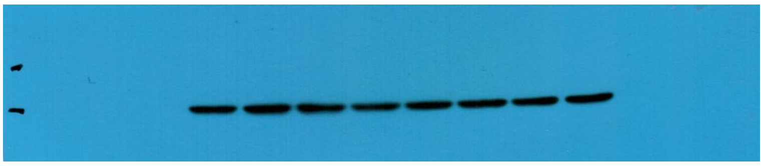

Figure 8F-5-β-actin
