# Supplementary material for: ATP7B R778L mutant hepatocytes resist copper toxicity by activating autophagy and inhibiting necroptosis
Source: Cell Death Discov. 2023 Sep 16;9:344. doi: 10.1038/s41420-023-01641-5 (PMC10505209; doi:10.1038/s41420-023-01641-5)
Supplement: Supplementary file 3 — Supplementary Table [file 41420_2023_1641_MOESM3_ESM.doc]

**Supplementary Table**

**Supplementary Table 1. The primers used in this work**

| **Primer** | **Forward** | **Reverse** |
| --- | --- | --- |
| Primer 1 | 5’-TCTAGAGCTAGCGAATTCGCCACCATGCCTGAGCAGGAGAGACAG -3’ | 5’- CCTTCTCAGCCACAGCAACCAC -3’ |
| Primer 2 | 5’-GTGGTTGCTGTGGCTGAGAAGG -3’ | 5’- GATGTACTGCTCCTCATCCCTGC -3’ |
| Primer 3 | 5’-GATGAGGAGCAGTACATCGACTACAAGGATGA -3’ | 5’-ATGATGATGATGCTCGAGTTATTTGTCGTCATCATC -3’ |
| Primer 4 | 5’-CCGGAATTCGCCACCATGGAGCCCGGCCGCGGCGGCACAG -3’ | CACCGTCATGGTCTTTGTAGTCGGCACAGATGCCAGTCAGCAGCGCCGAG -3’ |
| Primer 5 | CCGGAATTCGCCACCATGGAGCCCGGCCGCGGCGGCACAG -3’ | CTTGTAATCGATGTCATGATCTTTATAATCACCGTCATGGTCTTTGTAGT -3’ |
| Primer 6 | CCGGAATTCGCCACCATGGAGCCCGGCCGCGGCGGCACAG -3’ | CTAGACTAGTTCACTTGTCATCGTCATCCTTGTAATCGATGTCATGATC -3’ |
| Primer 7 | CCGGAATTCGCCACCATGTCGTCGGGCCTCCGCGCCGCTGA -3’ | CACCGTCATGGTCTTTGTAGTCGTACTGTGCCCACAGCACAGCTTTG -3’ |
| Primer 8 | CCGGAATTCGCCACCATGTCGTCGGGCCTCCGCGCCGCTGA -3’ | CTTGTAATCGATGTCATGATCTTTATAATCACCGTCATGGTCTTTGTAGT -3’ |
| Primer 9 | CCGGAATTCGCCACCATGTCGTCGGGCCTCCGCGCCGCTGA -3’ | CTAGACTAGTTCACTTGTCATCGTCATCCTTGTAATCGATGTCATGATC -3’ |
| Primer 10 | GATCCGGAGATGCAGATGAATGAAGCCTCGAGGCTTCATTCATCTGCATCTCCTTTTTG -3’ | AATTCAAAAAGGAGATGCAGATGAATGAAGCCTCGAGGCTTCATTCATCTGCATCTCCG -3’ |
| Primer 11 | GATCCGCAGAATGTTTGCAGACTATCCTCGAGGATAGTCTGCAAACATTCTGCTTTTTG -3’ | AATTCAAAAAGCAGAATGTTTGCAGACTATCCTCGAGGATAGTCTGCAAACATTCTGCG -3’ |
| Primer 12 | GATCCGCACAGAGCAATGTGTAATGACTCGAGTCATTACACATTGCTCTGTGCTTTTTG -3’ | AATTCAAAAAGCACAGAGCAATGTGTAATGACTCGAGTCATTACACATTGCTCTGTGCG -3’ |

**Supplementary Table 2. Interfering sequence specified for ATG16L1 gene**

| **Number** | **Sequence** |
| --- | --- |
| hATG16L1-1 | 5‘-GGAGATGCAGATGAATGAAGC-3’ |
| hATG16L1-2 | 5‘-GCAGAATGTTTGCAGACTATC-3’ |
| hATG16L1-3 | 5‘-GCACAGAGCAATGTGTAATGA-3’ |

**Supplementary Table 3. The Physico-chemical properties of the wild type (WT) and mutation type (R778L) ATP7B protein**

|  | **Molecular weight** | **Theoretical**  **PI** | **Total number of negatively charged residues** | **Total number of positively charged residues** | **The estimated half-life** | **Instability index** | **Aliphatic index** | **Grand average of hydropathicity (GRAVY)** |
| --- | --- | --- | --- | --- | --- | --- | --- | --- |
| **WT** | 157262.66 | 6.25 | 141 | 127 | 30h | 44.91 | 100.42 | 0.111 |
| **R778L** | 157219.63 | 6.21 | 141 | 126 | 30h | 44.68 | 100.68 | 0.117 |
